# Alternative NF-κB Signaling Discriminates Induction of the Tumor Marker Fascin by the Viral Oncoproteins Tax-1 and Tax-2 of Human T-Cell Leukemia Viruses

**DOI:** 10.3390/cancers14030537

**Published:** 2022-01-21

**Authors:** Stefanie Heym, Caroline F. Mohr, Hanna C. Engelbrecht, Bernhard Fleckenstein, Andrea K. Thoma-Kress

**Affiliations:** 1 FAU-Nachwuchsgruppe “Retroviral Pathogenesis” and BMBF Junior Research Group in Infection Research “Milk-Transmission of Viruses”, Institute of Clinical and Molecular Virology, Friedrich-Alexander-Universität Erlangen-Nürnberg (FAU), 91054 Erlangen, Germany; stefanie.heym@uk-erlangen.de (S.H.); hanna.engelbrecht@fau.de (H.C.E.); 2 Institute of Clinical and Molecular Virology, Friedrich-Alexander-Universität Erlangen-Nürnberg (FAU), 91054 Erlangen, Germany; caroline.mohr@freenet.de

**Keywords:** Fascin, tumor virus, HTLV-1, human T-cell leukemia virus type 1, alternative NF-κB, viral oncoprotein, Tax-1, Tax-2

## Abstract

**Simple Summary:**

The actin-bundling protein Fascin is upregulated in many types of cancers, including adult T-cell leukemia/lymphoma, a tumor induced by the oncogenic retrovirus human T-cell leukemia virus type 1 (HTLV-1). Transcriptional regulation of Fascin is heterogeneous between different cell types and tissues, and Fascin is usually expressed at low levels in T-cells. We have previously shown that a single viral oncoprotein, Tax-1 of HTLV-1, is a potent inducer of Fascin in T-cells depending on classical NF-κB signaling. In this study, we discovered that transcriptional activation of Fascin by viral oncoproteins depends on activity of both the classical and the alternative NF-κB signaling cascade. Comparisons between Tax-1 and Tax-2 from the closely related but non-oncogenic HTLV-2 revealed that alternative NF-κB signaling discriminates transcriptional induction of Fascin by the Tax proteins encoded by HTLVs. Together, Tax-1 and Tax-2 proteins are useful tools to study oncogenic signaling in T-cells.

**Abstract:**

Transcriptional regulation of the actin-bundling protein and tumor marker Fascin is highly diverse depending on cell and tumor type. Previously, we discovered that the viral oncoprotein Tax-1 of human T-cell leukemia virus type 1 (HTLV-1) considerably enhances Fascin expression in T-cells, depending on classical NF-κB signaling. In this study, we asked if the non-oncogenic Tax-2 of the related HTLV-2 is still able to induce Fascin by using luciferase assays, immunoblot, and qPCR. We found that Tax-2 only slightly induces Fascin expression compared to Tax-1; however, both Tax-1 and Tax-2 comparably activated a 1.6 kb fragment in the human *Fascin* promoter including Tax-responsive elements. Furthermore, we identified a link between Tax-induced activity of the alternative NF-κB pathway and Fascin induction. While treatment with the second mitochondria-derived activator of caspases (SMAC)-mimetic AZD5582, a compound known to robustly activate alternative NF-κB signaling, did not induce Fascin, combination of AZD5582 with activation of classical NF-κB signaling by Tax-2 significantly induced Fascin expression. In conclusion, our data demonstrate that both classical and alternative NF-κB activity are necessary for strong Fascin induction by the viral Tax oncoproteins, thus, shedding new light on the regulation of Fascin in T-cells and during viral transformation.

## 1. Introduction

Human T-cell leukemia virus type 1 (HTLV-1) was the first human oncogenic retrovirus to be discovered, which was isolated from a patient with cutaneous T-cell lymphoma [1]. Worldwide, there are at least five to ten million people that are HTLV-1 infected, and the virus is endemic in Japan, Sub-Saharan Africa, South America, the Caribbean, parts of the Middle East, Melanesia, and Central Australia [2]. In contrast, the distribution of the genetically closely related HTLV-2 is less well described. According to a rough estimation, approximately 800,000 people are infected, and HTLV-2 is endemic in the United States, Europe, South America, and Southeast Asia [3,4]. Other major discriminating factors between HTLV-1 and HTLV-2 are their different pathogenicity and cellular tropism. Roughly ten percent of HTLV-1-infected patients acquire clinical symptoms manifesting in HTLV-1-related illnesses as adult T-cell leukemia/lymphoma (ATLL) after several decades of latency or HTLV-associated inflammatory conditions such as HTLV-1-associated myelopathy/tropical spastic paraparesis (HAM/TSP) [1,5,6,7,8]. Although HTLV-2 was isolated from a patient with hairy cell leukemia, and HTLV-2 infection occurs with an increased lymphocyte count, it has no clear association with neoplasia [9,10,11,12,13]. Nevertheless, earlier observations ascertained an enhanced all-cause and cancer mortality of HTLV-2-infected patients [14]. Furthermore, multiple cases of neurodegenerative disorders of HTLV-2-infected patients and HAM/TSP-like symptoms have been described [15]. While HTLV-1 infects and transforms preferentially CD4^+^ T-cells in vivo, HTLV-2 is primarily detected in CD8^+^ T-cells [16,17]. However, HTLV-2 immortalizes peripheral blood mononuclear cells (PBMCs) and transforms both CD4^+^ and CD8^+^ T-cells in vitro [18,19]. Comparative studies between HTLV-1 and HTLV-2 have focused on uncovering the molecular mechanisms responsible for the differences in viral tropism, malignant transformation and clinical outcome in vivo [12].

The oncogenes driving T-cell transformation mediated by HTLV-1 and HTLV-2 are the viral transactivators Tax-1 or Tax-2, respectively. Among the Tax-2 proteins, Tax-2B of HTLV-2B is studied most extensively. Tax-1 and Tax-2 originate from the respective pX-region of the viral genome and share 85% sequence homology [12]. However, there are two major molecular differences described for Tax-1 and Tax-2: (1) Tax-1 harbors a postsynaptic density protein (PSD95), Drosophila disc large tumor suppressor (DlgA), and zonula occludens-1 protein (ZO-1) (PDZ) binding motif (PBM) at its C-terminus; the PBM is essential for the interaction of Tax-1 with host cell factors regulating cell cycle progression and tumor suppression [20,21]. Furthermore, the PBM of Tax-1 was proposed to be essential for induction of interleukin-2 independent growth of T-cells, and the use of animal models has revealed a significant role of the PBM for viral persistence and cellular proliferation during HTLV-1 infections [21,22,23,24,25]. (2) Tax-1 contains a leucine zipper-like motif unreported in Tax-2, which is crucial for the activation of the alternative nuclear factor kappa-light-chain-enhancer of activated B cells (NF-κB) signaling pathway [22,26]. Both the PBM and the induction of the alternative NF-κB pathway are critical for the transforming activity of Tax-1 and may be the essential factors to discriminate the transforming potential of Tax-1 from that of Tax-2 in vivo.

Two pathways of NF-κB signaling have been described, the classical or canonical, and the alternative or non-canonical signaling cascade. Hereby, DNA binding proteins of the NF-κB/Rel protein family, RelA, RelB, c-Rel, p105/p50, and p100/p52, are involved. They form homo- and heterodimers, which after translocation to the nucleus, induce the expression of NF-κB target genes involved in immune modulation, development, cell proliferation, and survival. In the classical NF-κB pathway, the nuclear translocation of the RelA:p50 heterodimer is blocked by its binding to the NF-κB inhibitor (IκB). Release of this inhibition is regulated by the inhibitor of kappa B kinase (IKK)-complex, formed by the two kinase subunits IKK1 and IKK2 and the regulatory subunit NF-κB essential modulator (NEMO; IKK3) [27,28,29]. Both, Tax-1 and Tax-2 interact with NEMO to activate classical NF-κB signaling [30,31]. The activity of alternative NF-κB signaling is initiated by tumor necrosis factor receptor (TNFR) superfamily members such as CD40, lymphotoxin-β receptor, or B-cell activating factor (BAFF) receptor and depends on stabilization and accumulation of the NF-κB inducing kinase (NIK), which is achieved by destabilization of a complex of TNFR-associated factor (TRAF) and cellular inhibitor of apoptosis (cIAP) [28,32]. NIK leads to activation of the IKK-complex of the alternative NF-κB pathway, which consists of IKK1 homodimers, followed by p100 phosphorylation and processing of p100 to p52. Afterward, the RelB:p52 heterodimer can translocate to the nucleus in order to induce target gene expression [33]. Tax-1 can activate the alternative NF-κB signaling cascade independent of NIK by recruiting NEMO to the alternative IKK-complex [34]. The region between amino acid (aa) 225 and 232 of Tax-1, which contains the unique leucine zipper-like motif not present in Tax-2, is responsible for inducing alternative NF-κB activity [26]. Therefore, activation of the alternative NF-κB pathway by Tax-2 is considered absent. Yamagishi and colleagues have identified another NIK-dependent regulation mechanism of alternative NF-κB induction in ATLL. Thereby, epigenetic changes regulated by Polycomb group proteins result in an increased abundance of NIK in ATLL-derived cells via negative regulation of the micro-RNA miR-31 [35].

By stimulation of NF-κB signaling, Tax-1 induces the expression of several cellular target genes, among them being the actin-bundling protein Fascin [36]. Fascin is naturally expressed at high levels in dendritic, neuronal, mesenchymal, and endothelial cells and is typically responsible for the stabilization of bundled actin filaments [37,38]. Furthermore, Fascin expression is upregulated in many types of cancer, e.g., in breast, lung, colon, and skin cancer, and has therefore gained interest as a potential tumor marker. However, the regulation of *Fascin* transcription varies depending on cell and tumor type [39,40]. In normal T lymphocytes, *Fascin* expression occurs at low levels. Nevertheless, we previously observed a strong upregulation in HTLV-1 transformed as well as ATLL-derived CD4^+^ T-cells [36]. In addition, we have identified a two-way mechanism of Fascin regulation by Tax-1 depending on the classical NF-κB pathway and the *Fascin* promoter and a second regulatory mechanism independent of the promoter but sensitive to the Src family kinase inhibitor PP2 [41]. In the past, Fascin displayed an association with aggressiveness and infiltrative potential of several carcinomas, and we could previously show that knockdown of Fascin reduces the invasion of ATLL-derived cells through extracellular matrix [36]. Recently, we also found that both Tax-induced and endogenous Fascin is important for HTLV-1 release and cell-to-cell transmission [42].

In the present study, we evaluate whether Tax-2, the close but non-oncogenic relative of Tax-1, is also able to induce Fascin, focusing on Tax-2B of HTLV-2B. Tax-1-mediated Fascin induction is NF-κB dependent [41]. Importantly, Tax-1 is a potent enhancer of both classical and alternative NF-κB signaling, while Tax-2 is reported to only activate the classical NF-κB pathway [26]. Therefore, we aim to analyze if different NF-κB activatory capacities can further unravel the regulation of Fascin in T-cells. Here, we demonstrate for the first time that both classical and alternative NF-κB signaling are involved in Fascin induction by viral oncoproteins. At the same time, the activation level of the alternative NF-κB signaling cascade by Tax proteins is linked to the level of Fascin expression. In conclusion, we show that comparative studies of Tax-1 and Tax-2 are useful to further enlighten the regulatory mechanisms involved in upregulation of Fascin in T-cells during viral transformation.

## 2. Materials and Methods

### 2.1. Cell Culture

The CD4^+^ T-cell lines Jurkat (from acute lymphoblastic leukemia) [43], Molt-4 [44], MT-2 (HTLV-1 in vitro transformed) [45], Tesi (Tax-1-transformed) [46], the Jurkat-derived cell line SVT35 (carrying an NF-κB-driven CD14 reporter gene) [47], and the HTLV-2-transformed T-cell lines MoT [48] and C3-44-Mo [49] were cultured in RPMI 1640M with L-glutamine (0.35 g/L), penicillin/streptomycin, and 20% (Tesi) or 10% (all other cell lines) fetal calf serum. For cultivation of Jurkat, SVT35, Molt-4, and Tesi, media were supplemented with 45% Panserin 401 (PAN-Biotech, Aidenbach, Germany), and Tesi cells were additionally cultured in media supplemented with 40 U/mL interleukin-2 (Roche Diagnostics GmbH, Mannheim, Germany).

### 2.2. Plasmids and Cloning

Luciferase reporter constructs of the human *Fascin* promoter phF1.6 (−1499 to +123) and phF-TRR carrying a Tax-1-responsive *Fascin* promoter fragment (−1499 to −1325) upstream of the *Fascin* core promoter (−88 to +123) have been described previously [41,50]. Expression plasmids for Tax-1, Tax-2 (encoding Tax-2B from HTLV-2B), and Tax-1/Tax-2 chimeras (in pEF-1α) and the control plasmid pEF-1α were kindly provided by M. Higuchi and M. Fujii [26]. J.M. Peloponese kindly provided expression plasmids for Tax-1 (pCAG-FLAG-Tax-1) and the truncation mutant TaxTD319 [51]. pcTax-2F (encoding Tax-2B from HTLV-2B) contains an internal FLAG-6His tag between aa position 337 and 338 of Tax-2B and was a kind gift from the late U. Bertazzoni, Verona, Italy [52]. Sequences of the two different Tax-2B expression constructs pEF-Tax-2 and pcTax-2F were analyzed by automated sequencing using the primers Tax2-fwd-601: 5′-CACACAGGAGCGGTCATAGT-3′, Tax2-rev-801: 5′-AGAGAGGATTGAACTAC-3′, and Tax2-rev-401: 5′-AGGTGGTGTAGATGTTTTGG-3′. Evaluation of sequences was performed using *SnapGene software* (GSL Biotech LLC, San Diego, CA, USA). pcTax, a plasmid for wt-Tax-1 driven by a cytomegalovirus (CMV) promoter [53], pIκBα-DN, encoding a dominant-negative inhibitor of IκBα [54], pcDNA3.1 (pcDNA; Life Technologies GmbH, Darmstadt, Germany), and pGL3-Basic, the luciferase-reporter vector without eukaryotic promoter and enhancer sequences (Promega, Mannheim, Germany), were used.

### 2.3. Inhibitors

The IKK2 inhibitor ACHP (2-Amino-6-(2-(cyclopropylmethoxy)-6-hydroxyphenyl)-4-(4-piperidinyl)-3-pyridinecarbonitrile) (Merck, Darmstadt, Germany), the NFAT-inhibitor cyclosporine A (CsA; cyclo-(L-Alanyl-D-alanyl-N-methyl-L-leucyl-N-methyl-L-leucyl-N-methyl-L-valyl-3-hydroxy-N,4-dimethyl-L-2-amino-6-octenoyl-L-a-amino-butyryl-N-methylglycyl-N-methyl-L-leucyl-L-valyl-N-methyl-L-leucyl)) (Biomol, Hamburg, Germany), and the Src-family-kinase inhibitor PP2 (4-Amino-5-(4-chlorophenyl)-7-(t-butyl)pyrazolo[3,4-d]pyrimidine) (Merck) were dissolved in DMSO. Inhibitors were used for 48 h at the indicated concentrations. To inhibit mRNA transcription, cells were treated with 5 µg/mL actinomycin D (Sigma-Aldrich, St. Louis, MO, USA) dissolved in DMSO, 24 h after transfection with pEF-Tax-1, pEF-Tax-2, or pEF-1α for a time course of 0, 1, 2, 4, and 8 h. The SMAC-mimetic AZD5582 (MedChemExpress, South Brunswick Township, NJ, USA) was dissolved in DMSO, and cells were treated with concentrations of 1, 10, 100, and 500 nM AZD5582 for 24 or 48 h. The SMAC-mimetic Birinapant (TL32711; Selleckchem, Houston, TX, USA) was dissolved in DMSO, and cells were treated with 10 nM or 50 nM for 24 h.

### 2.4. Transfections

Transient transfections of CD4^+^ T-cells (Jurkat, SVT35, or Molt-4) were performed by electroporation as described [55] using a total amount of either 50 or 100 μg plasmid DNA to transfect 5 × 10^6^ or 1 × 10^7^ cells, respectively. In experiments where NF-κB signaling was blocked, 10 μg of pIκBα-DN were used. Cells were harvested 48 h after transfection.

### 2.5. Luciferase Assay

Jurkat T-cells (5 × 10^6^ cells) were transfected by electroporation using 20 µg of reporter plasmids and 30 µg of the indicated expression plasmids. Experiments were performed in technical triplicates and harvested 48 h after transfection. After lysis of cells, luciferase activity was measured using an *Orion Microplate Luminometer* (Berthold, Pforzheim, Germany) as described [55]. Relative light units (RLU) of the technical triplicates were normalized to the respective protein concentrations determined by Bradford assay (*Roti*^®^*Quant*, Carl Roth, Karlsruhe, Germany). The means of at least three independent experiments are depicted.

### 2.6. Immunoblots

Cells were lysed and immunoblot was performed as described [55]. In addition to *freeze-and-thaw* cycles, samples were sonicated three times for 20 s. Immunoblots were probed using the following primary antibodies: rabbit monoclonal antibodies anti-NF-κB2 (p100/p52; 18D10; Cell Signaling Technology, Danvers, MA, USA), mouse monoclonal antibodies anti-Fascin (55K-2; Dako Deutschland GmbH, Hamburg, Germany), anti-FLAG (M2; Sigma-Aldrich), anti-β-actin (ACTB; AC-15; Sigma-Aldrich), anti-HSP90α/β (F8; Santa Cruz Biotechnology, Dallas, TX, USA), and mouse anti-Tax-1. The latter were derived from the hybridoma cell line 168B17-46-34, provided by B. Langton through the AIDS Research and Reference Reagent Program, Division of AIDS, NIAID, NIH [56]. Tax-2 specific antibodies were obtained after immunizing rabbits with the synthetic peptide CAAQGESSTQKVRPSHTNNPK containing the 20 C-terminal amino acids of Tax-2B (Exbio, Vestec, Czech Republic) according to a previous study [52]. Secondary antibodies conjugated with horseradish peroxidase were obtained from GE Healthcare (Little Chalfont, UK). Enhanced chemiluminescence was detected using *Advanced Fluorescence Imager camera* (*ChemoStar*, Intas Science Imaging GmbH, Göttingen, Germany) and representative immunoblots are shown. To quantify the amount of protein, densitometric analysis was performed either for a representative immunoblot or of at least three independent experiments using *Advanced Image Data Analyser* (*AIDA Version 4.23*, Raytest Isotopenmessgeräte GmbH, Straubenhardt, Germany). p100 processing was determined by dividing the amount of p52 protein by the total amount of p100 and p52 proteins (p100 processing = p52/(p100 + p52). Alternatively, p52 expression was determined by normalizing the amount of p52 on the respective housekeeping gene and on values obtained from mock transfected cells. Original images of the immunoblots can be found in the supplementary file.

### 2.7. Flow Cytometry

The cell surface marker CD14 in transfected SVT-35 cells was stained using monoclonal antibodies anti-CD14-PE-Cy5 (1 h; Immunotools, Friesoythe, Germany). To evaluate toxicity of AZD5582, vitality of Jurkat T-cells was detected upon staining with 10 µM propidium iodide (PI) immediately prior to the measurement. As a toxicity control, cells were treated with 15 µM of the topoisomerase II inhibitor etoposide for 48 h (Sigma-Aldrich). Flow cytometry analysis was performed using a *BD LSRII flow cytometer* (BD Bioscience, Heidelberg, Germany).

### 2.8. Quantitative Real-Time RT-PCR (qPCR)

To quantitate Fascin copy numbers, total cellular RNA was isolated (*NucleoSpin*^®^ *RNA*, Macherey Nagel, Düren, Germany) and reversely transcribed to cDNA using *random hexamer primers* and *Superscript^TM^ II Reverse Transcriptase* (both Thermo Fisher Scientific, Waltham, MA, USA) according to manufacturers’ instructions. Thereafter, quantitative real-time RT-PCR (qPCR) of Fascin and ß-actin (ACTB) transcripts was performed in technical triplicates using *TaqMan*^®^ *Universal PCR Master Mix* (Applied Biosystems, Foster City, CA, USA) as described [55]. Relative copy numbers (rcn) of *Fascin* were determined by normalizing the copies of *Fascin* on those of *ß-actin*. At least three independent experiments were performed as indicated.

### 2.9. Statistics

For statistical analyses, Student’s *t* test (unpaired) and two-way analysis of variance (ANOVA) were performed using *Microsoft Office Excel*. *p* values < 0.05 were considered as significant (*).

## 3. Results

### 3.1. Tax-2 Slightly but Significantly Induces Fascin Expression and Alternative NF-κB Signaling in T Lymphocytes

**Figure 1:**
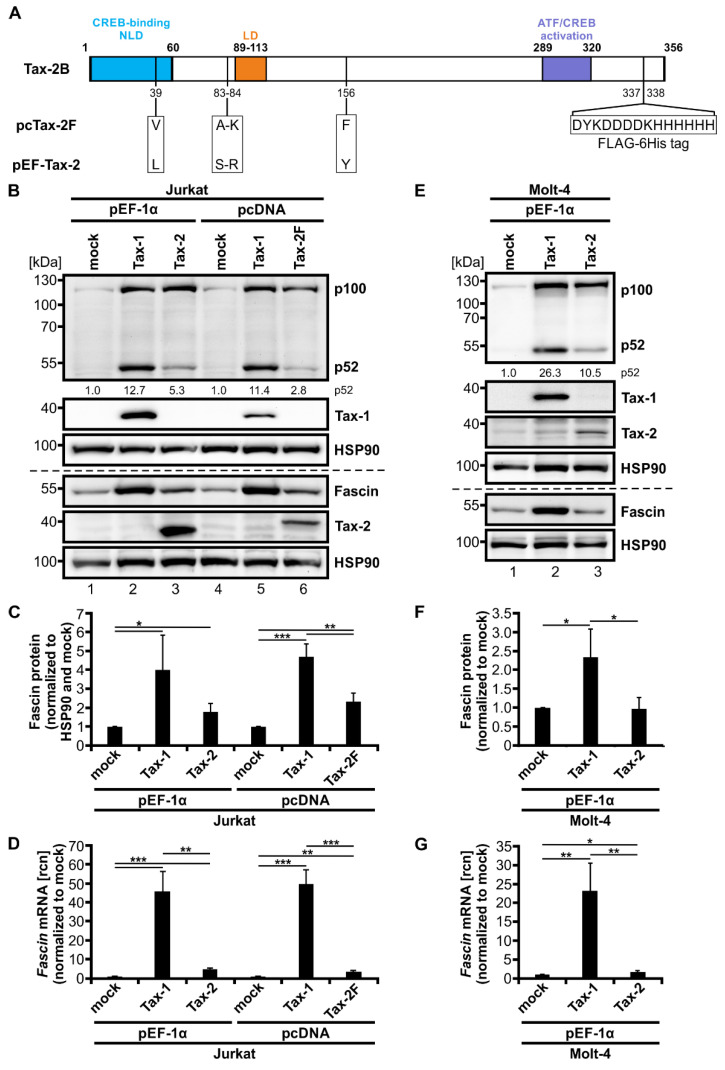
Tax-2 induces both Fascin expression and alternative NF-κB signaling but is less potent than Tax-1. (**A**) Scheme of Tax-2B wildtype (wt) and sequence analysis of the two Tax-2B expression constructs pcTax-2F (with internal FLAG-6His tag) and pEF-Tax-2 (untagged). Numbers indicate amino acid (aa) positions of Tax-2B wt, which consist of several domains: a cAMP response element-binding protein (CREB)-binding motif (aa 1-60) harboring a nuclear localization determinant (NLD), an additional localization domain (LD; aa 89-113), and an activating transcription factor (ATF)/CREB activation domain (aa 289-320). Differences between pcTax-2F and pEF-Tax-2 sequences are indicated. (**B**–**D**) Jurkat or (**E**–**G**) Molt-4 T-cells were transfected with pEF-Tax-1, pEF-Tax-2, pcTax-1, pcTax-2F, or the respective mock vectors pEF-1α or pcDNA as indicated (100 µg each). (**B**,**E**) Representative immunoblot of NF-κB2 (p100 and p52), Tax-1, Fascin, Tax-2, and the housekeeping gene heat shock protein 90 (HSP90). Values depict densitometric analysis of p52 protein normalized to HSP90 and the respective mock vector of the representative immunoblot. (**C**,**F**) Densitometric analysis of Fascin protein normalized to HSP90 and the respective mock vector. Mean values of three or four independent experiments ± SD were compared using Student’s *t* test (* *p* < 0.05, ** *p* < 0.01, *** *p* < 0.001). (**D**,**G**) *Fascin* transcript levels were measured by quantitative PCR (qPCR) and normalized to *β-actin*. Resulting relative copy numbers (rcn) were normalized to the respective mock vector and the mean of three independent experiments ± SD were compared using Student’s *t* test (* *p* < 0.05, ** *p* < 0.01, *** *p* < 0.001).

The oncoprotein Tax-1 has been identified to be a potent inducer of Fascin depending on the activity of the classical NF-κB signaling pathway [36,41]. To gain more insights into transcriptional regulation of *Fascin* in T-cells, we evaluated if the closely related protein Tax-2 of the non-oncogenic HTLV-2 possesses the same properties. For this purpose, Jurkat T-cells were transfected with different Tax-1 and Tax-2 expression plasmids, (1) either controlled by a CMV-promoter (pcTax-1; pcTax-2F carrying a C-terminally integrated FLAG-6His tag), or (2) by the elongation factor 1 α promoter (EF-1α; pEF-Tax-1; pEF-Tax-2). Both Tax-2 expression constructs correspond to Tax-2B from HTLV-2B. The *tax-2B* coding sequence of the pcTax-2F construct (GenBank accession number: DQ022075) was originally isolated from PBMCs of an Italian patient infected with HTLV-2B [57]. In contrast, the coding sequence of the pEF-Tax-2 plasmid (GenBank accession number: AF292001) originated from a North American intravenous drug user [58]. Automated sequencing of the *tax-2B* coding sequences of pcTax-2F and pEF-Tax-2B and sequence alignment (Figure 1A) displayed several polymorphisms resulting in different Tax-2B aa sequences at positions 39, 83, 84, and 156. As expected, expression analysis of the different Tax constructs in Jurkat T-cells revealed that Tax-1 considerably enhanced p100 expression and processing in Jurkat T-cells independent of the promoter used. Additionally, p100 processing into p52 was quantified by densitometric analysis of the representative immunoblot (Figure 1B, pEF-Tax-1: 12.7-fold, pcTax-1: 11.4-fold induction of p52 protein compared to mock), representing activity of the alternative NF-κB signaling pathway. Independent of the differences in the coding sequence, transient transfection of pEF-Tax-2 and pc-Tax-2F plasmids strongly induced p100 expression, thus corresponding to the activity of the classical NF-κB pathway and confirming earlier work [22,26]. To our surprise, transfection of Tax-2 expression constructs slightly increased p52 expression, albeit at much lower levels than those displayed in presence of Tax-1 (pEF-Tax-2: 5.3-fold, pc-Tax-2F: 2.8-fold induction of p52 protein compared to mock). This contradicts previous findings that claimed absence of alternative NF-κB signals in presence of Tax-2 [22,26]. In contrast to Tax-1, which led to a substantial induction of Fascin protein (Figure 1C; *p* < 0.05) and *Fascin* transcripts (Figure 1D; *p* < 0.001), Tax-2 only slightly but significantly enhanced Fascin protein (*p* < 0.05) and *Fascin* transcripts (*p* < 0.01) independent of the aa discrepancies as well as the promoter used. Thus, our data show that Tax-2 not only induces classical but also alternative NF-κB signaling in Jurkat T-cells, leading to the hypothesis that alternative NF-κB activity is required for induction of Fascin expression.

To further consolidate results, we additionally performed transient transfection of Molt-4, another CD4^+^ T-cell line, with pEF-1α expression vectors for Tax-1 and Tax-2B (Figure 1E–G). The basal activity of the classical and alternative NF-κB signaling pathway was comparable between mock-transfected Molt-4 T-cells (Figure 1E) and mock-transfected Jurkat T-cells (Figure 1B), resulting in comparable levels of p100 and p52 protein, respectively. Confirming data from Jurkat T-cells, both Tax-1 and Tax-2 enhanced p100 expression similarly, resembling classical NF-κB signaling. They both induced processing of p100 to p52; however, Tax-1 was more potent than Tax-2 in increasing alternative NF-κB signaling in Molt-4 T-cells (pEF-Tax-1: 26.3-fold; pEF-Tax-2: 10.5-fold induction of p52 protein compared to mock). Quantification of Fascin protein (Figure 1F, *p* < 0.05) and *Fascin* transcript (Figure 1G, *p* < 0.01) expression revealed that Tax-1 is also a potent, significant inducer of Fascin in Molt-4 T-cells, although the enhancement was lower than observed in Jurkat T-cells (Figure 1C,D). Tax-2 was able to slightly but significantly increase *Fascin* transcripts in Molt-4 T-cells (Figure 1G, *p* < 0.05); however, different than in Jurkat T-cells (Figure 1B,C), Tax-2 did not lead to the induction of Fascin protein expression in Molt-4 cells (Figure 1E,F). In conclusion, Tax-2 is a potent inducer of Fascin expression independent of the promoter used but with varying effects in different T-cell lines. Together, these data make comparisons between Tax-1 and Tax-2 valuable tools to study transcriptional regulation of viral target genes in T-cells.

### 3.2. A 1.6 kb Fragment of the Fascin Promoter Is NF-κB-Dependently Upregulated by Tax-2 and Tax-1 at Comparable Levels

**Figure 2:**
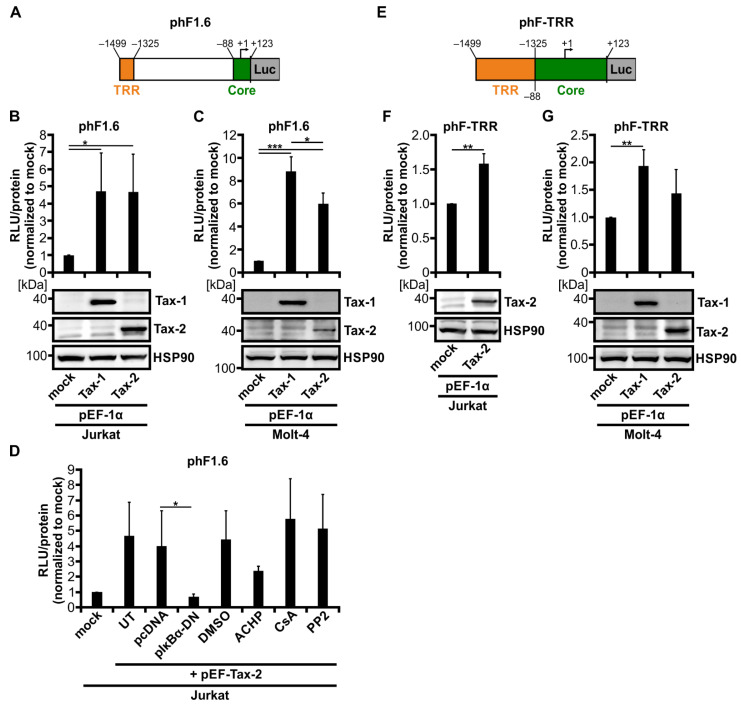
Tax-2 and Tax-1 activate a 1.6 kb fragment of the human *Fascin* promoter at comparable levels. (**A**) Schematic representation of the luciferase (Luc) reporter construct phF1.6 carrying the 1.6 kb fragment of the human *Fascin* promoter. The positions of the Tax-responsive region (TRR) and of a core promoter fragment (Core) are indicated. Numbers show distances from the transcription start point (+1) of the full-length human *Fascin* promoter. (**B**,**D**) Jurkat or (**C**) Molt-4 T-cells were co-transfected with phF1.6. After 48 h, luciferase activity was measured (RLU, relative light units) followed by normalization on total protein content and on mock-transfected samples (pEF-1α). The mean values of three independent experiments ± SD are shown and were compared using Student’s t test (* *p* < 0.05, *** *p* < 0.001). (**B**,**C**) Cells were co-transfected with phF1.6 (20 µg), pEF-Tax-1 (Tax-1), pEF-Tax-2 (Tax-2), or the mock control vector pEF-1α (30 µg each). Immunoblots of Tax-1, Tax-2, and the housekeeping gene heat shock protein 90 (HSP90) are shown. (**D**) Cells were co-transfected with phF1.6 (20 µg) and expression plasmids pEF-Tax-2 (Tax-2) (20 µg) and pIκBα-DN (10 µg), or the respective control vectors pEF-1α or pcDNA. After co-transfection with pEF-Tax-2, cells were treated with 10 µM ACHP, 1 µg/mL cyclosporine A (CsA), 5 µM PP2, or the solvent control dimethyl sulfoxide (DMSO) for 48 h, or were left untreated (UT). (**E**) Schematic representation of the Luc reporter construct phF-TRR carrying the TRR of the human *Fascin* promoter inserted upstream of the *Fascin* core promoter. Numbers show distances from the transcription start point (+1) of the full-length human *Fascin* promoter. (**F**) Jurkat or (**G**) Molt-4 T-cells were co-transfected with phF-TRR (20 µg), pEF-Tax-1 (Tax-1), pEF-Tax-2 (Tax-2), or the mock control vector pEF-1α (30 µg each) as indicated. After 48 h, luciferase activity was measured (RLU) and normalized to total protein content and to mock-transfected samples (pEF-1α). Mean values of three independent experiments ± SD are shown and were compared using Student’s t test (** *p* < 0.01). Immunoblots of Tax-1, Tax-2, and HSP90 are depicted.

Since an earlier work from our group has indicated that the 1.6 kb fragment of the human *Fascin* promoter (phF1.6) is regulated by Tax-1 via the classical NF-κB pathway [41], and Tax-2 is also a potent inducer of this pathway [22,26], we first checked whether Tax-2 is also able to transactivate the Tax-responsive promoter region phF1.6 (Figure 2A). Therefore, Jurkat T-cells were co-transfected with phF1.6 and Tax-2 or Tax-1 as a positive control. Although Tax-2 was only slightly able to foster *Fascin* transcription in CD4^+^ T-cell lines (Figure 1D,G), Tax-2 could transactivate phF1.6 in Jurkat T-cells comparable to Tax-1 (Figure 2B; *p* < 0.05) and was also expressed in similar amounts as Tax-1 (Figure 2B). The activity of phF1.6 in Molt-4 T-cells was also significantly enhanced by Tax-2 (Figure 2C; *p* < 0.001), but in contrast to Jurkat T-cells, the induction was significantly lower than by Tax-1 (*p* < 0.05), which may be due to lower expression levels of Tax-2 than of Tax-1 in Molt-4 cells (Figure 2C). In general, the transactivation of phF1.6 by the Tax oncoproteins was slightly higher in Molt-4 T-cells than in Jurkat T-cells.

To determine whether transactivation of the human *Fascin* promotor depends on classical NF-κB signaling induced by Tax-2 comparable to what we have ascertained earlier with Tax-1 [41], Jurkat T-cells were co-transfected with phF1.6 and Tax-2 in the presence of NF-κB inhibitors (Figure 2D). Co-transfection of the dominant-negative NF-κB inhibitor α (IκBα; pIκBα-DN) or addition of the IKK2 inhibitor ACHP (10 µM) led to reduced Tax-2-mediated transactivation of phF1.6 (*p* < 0.05; not significant). In contrast, neither the nuclear factor of activated T-cells (NFAT) inhibitor cyclosporine A (CsA) nor the Src family kinase inhibitor PP2 affected the Tax-2 mediated activation of the promoter, paralleling previous findings obtained with Tax-1 [41]. We previously identified a Tax-responsive region (TRR) within phF1.6 (phF-TRR; Figure 2E) [41], which carries similarities to viral cAMP response element (vCRE) sites in the viral LTR promoter region [59,60]. To test whether phF-TRR is also sensitive to Tax-2, Jurkat or Molt-4 T-cells were co-transfected with luciferase reporter constructs phF-TRR and Tax-2 expression plasmids. Not only Tax-1, as described earlier in Jurkat T-cells [41], but also Tax-2 significantly enhanced transactivation of phF-TRR by 1.6-fold (Figure 2F; *p* < 0.01). In Molt-4 T-cells, however, the observed induction of phF-TRR activity by Tax-2 was visible but was not significant despite expression of Tax-2 (Figure 2G). Taken together, these results clearly illustrate that not only Tax-1 but also Tax-2 can activate the *Fascin* promoter phF1.6 relying on NF-κB activity. Nevertheless, the strength of phF-TRR activation depends on the cell line used and the expression levels of Tax proteins. Since only Tax-1 leads to a strong induction of *Fascin* transcription compared to Tax-2, both the 1.6 kb fragment phF1.6 and the TRR seem to be necessary, but not sufficient to regulate oncoprotein-mediated Fascin expression.

### 3.3. Stability of Fascin Transcripts Is Neither Enhanced by Tax-1 nor by Tax-2

**Figure 3:**
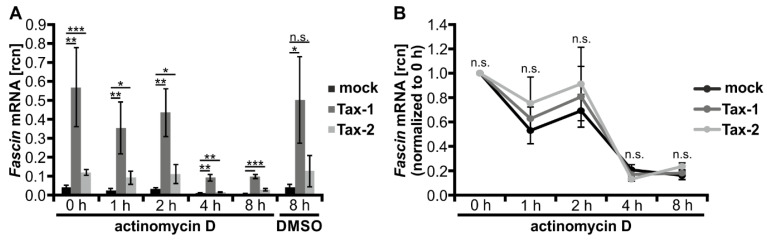
Tax-1 and Tax-2 do not stabilize *Fascin* transcripts. (**A**,**B**) Jurkat T-cells were transfected with expression plasmids pEF-Tax-1 (Tax-1), pEF-Tax-2 (Tax-2), or the mock control vector pEF-1α (100 µg each). After 24 h, cells were treated with 5 µg/mL actinomycin D for a time course of 0, 1, 2, 4, and 8 h. *Fascin* transcript levels were measured by quantitative PCR (qPCR) and normalized to *β-actin*, resulting in relative copy number (rcn) of *Fascin* transcripts. The means of three independent experiments ± SD are shown. (**A**) Cells treated with dimethyl sulfoxide (DMSO) for 8 h served as solvent control. Values were compared using Student’s t test (* *p* < 0.05, ** *p* < 0.01, *** *p* < 0.001, n.s., not significant). (**B**) Rcn were normalized to the respective time point t = 0 h of actinomycin D treatment, and two-way ANOVA was performed (n.s., not significant).

Since phF-TRR and phF1.6 are not decisive for the strong, Tax-1-induced *Fascin* transcription, we examined whether Tax-1 enhances the stability of *Fascin* mRNA compared to Tax-2. For this purpose, Jurkat T-cells were transfected with the pEF-1α expression vectors for Tax-1 or Tax-2, or the mock vector pEF-1α. Starting at 24 h post transfection, cells were treated with the inhibitor of RNA synthesis actinomycin D, and time course experiments were performed. In line with earlier results (Figure 1), Tax-1 strongly and Tax-2 moderately but significantly induced *Fascin* transcript expression independent of the time point of actinomycin D treatment (Figure 3A). For a better evaluation of transcript stability during actinomycin D treatment, relative copy numbers (rcn) of transfected cells were normalized to the respective time point t = 0 h (24 h post transfection; Figure 3B). All three transfection conditions led to a decline in *Fascin* transcript levels, and no significant differences in *Fascin* mRNA stability were observed between Tax-1, Tax-2, and mock-transfected cells at any time point. In conclusion, these data exclude stabilizing effects of Tax-1 on *Fascin* transcripts that could explain the different capabilities of Tax-1 and Tax-2 in inducing Fascin expression.

### 3.4. The PDZ Binding Motif of Tax-1 Is Dispensable for Transcriptional Induction of Fascin

**Figure 4:**
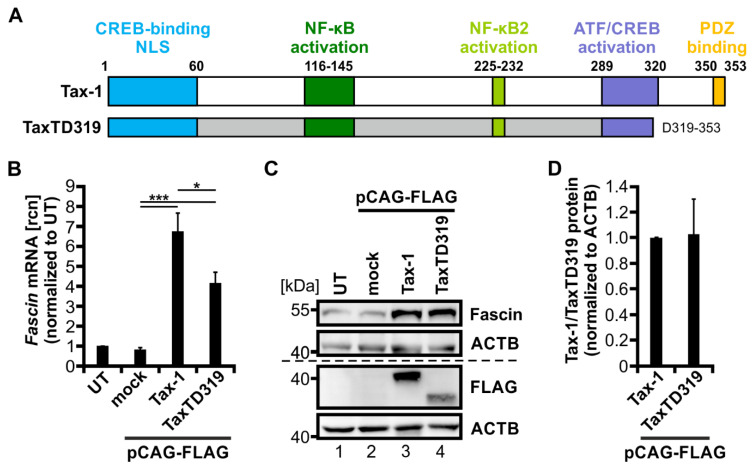
The PDZ binding motif of Tax-1 is necessary for full transcriptional induction of *Fascin*. (**A**) Scheme of Tax-1 wildtype (wt) protein (amino acid (aa) 1-353) and TaxTD319 deletion mutant (aa 1-319). Protein domains of Tax-1 are indicated: a cAMP response element-binding protein (CREB)-binding motif (aa 1-60) harboring a nuclear localization sequence (NLS), an NF-κB activation domain (aa 116-145), an NF-κB2 activation domain (aa 225-232), an activating transcription factor (ATF)/CREB activation domain (aa 289-320), and a PDZ binding motif (aa 350-353). (**B**,**C**) Jurkat T-cells were transfected with FLAG-tagged constructs for Tax-1, the deletion mutant TaxTD319, or the mock control vector pCAG-FLAG (50 µg), or were left untreated (UT). (**B**) After 48 h, *Fascin* transcript levels were measured by quantitative PCR (qPCR) and normalized to those of *β-actin*. Relative copy numbers (rcn) were normalized to UT, and the means of four independent experiments ± SE were compared using Student’s t test (* *p* < 0.05, *** *p* < 0.001). (**C**) Representative immunoblot of Fascin, Tax (detected by FLAG antibodies), and β-actin (ACTB). (**D**) Densitometric analysis of FLAG-labeled protein normalized to ACTB and Tax-1 wt. Mean values of three independent experiments ± SE were compared using Student’s t test.

To further uncover the differences between Tax-1 and Tax-2 that account for the heterogeneity in transcriptional regulation of *Fascin*, we tested whether a crucial domain that is present in Tax-1 but not in Tax-2 may account for the observed phenotypes: the PBM. While Tax-1 harbors a C-terminal PBM (Figure 4A; aa 350-353; ETEV), this motif is absent in Tax-2 (Figure 1A). Briefly, the PBM has been shown to interact with proteins containing a PDZ domain. In the context of Tax-1, this interaction is important for viral persistence and cell proliferation, thereby modulating T-cell transformation [21,23,25]. To check the hypothesis of whether the PBM of Tax-1 is also required for transcriptional induction of *Fascin*, Jurkat T-cells were transfected with plasmid TaxTD319 lacking aa 319-353 of Tax-1 including the PBM and were compared to cells transfected with wildtype Tax-1 (Tax wt), the mock vector (pCAG-FLAG), or untreated cells (UT). qPCR analysis (Figure 4B) revealed that TaxTD319 is still able to significantly enhance *Fascin* transcripts four-fold compared to mock (*p* < 0.001), but to a significantly lesser extent than wildtype Tax-1 (7-fold; *p* < 0.05). Induction of Fascin expression could be further confirmed by immunoblot (Figure 4C). Since TaxTD319 is still able to increase Fascin while being expressed at comparable levels such as Tax-1 wt (Figure 4D), the PBM of Tax-1 is dispensable for the regulation of Fascin; however, it contributes to full transcriptional induction of *Fascin* expression.

### 3.5. Fascin Induction by Tax-1/Tax-2 Chimeras and by HTLV-2 Correlates with Activity of the Alternative NF-κB Signaling Pathway

**Figure 5:**
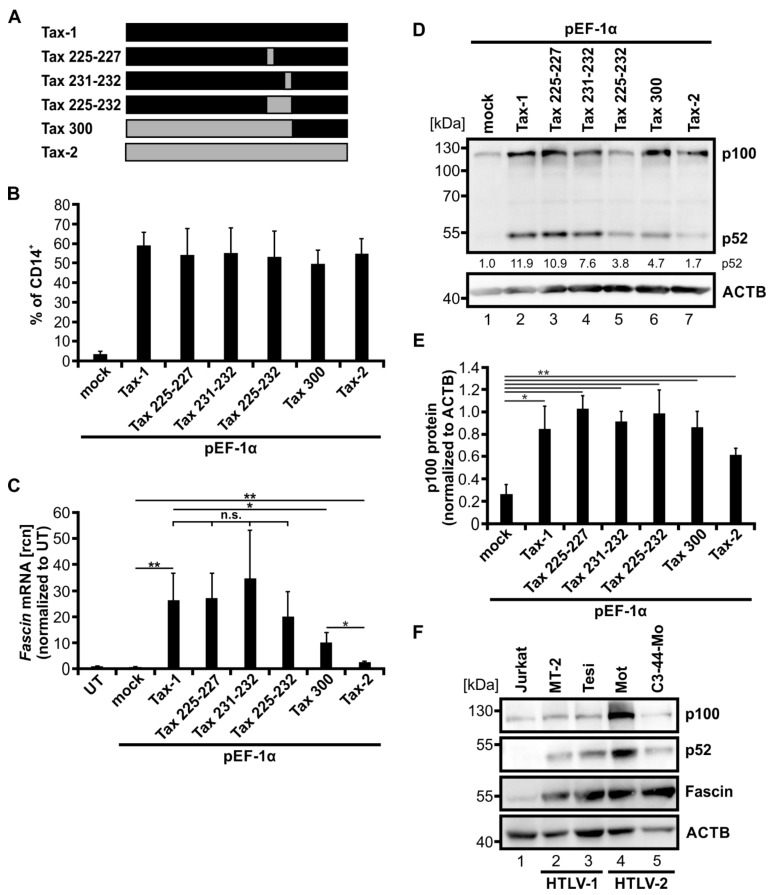
Fascin induction by Tax-1/Tax-2 chimeras and in HTLV-2-transformed T-cells correlates with activity of the alternative NF-κB signaling pathway. (**A**) Scheme of Tax-1, Tax-2, and Tax-1/Tax-2 chimeras. Exchanged amino acids in the respective mutants Tax 225-227, Tax 231-232, Tax 225-232, and Tax 300 are indicated. Black and grey bars show fragments originating from Tax-1 and Tax-2, respectively. (**B**) SVT35 Jurkat T-cells carrying an NF-κB-dependent CD14-reporter were transfected with either pEF-Tax-1, pEF-Tax-2, the listed plasmids encoding chimeras of both proteins, or the mock control vector pEF-1α (50 µg). NF-κB-driven CD14 surface expression was detected at 48 h after transfection by flow cytometry using anti-CD14 antibodies. Mean values ± SD of three independent experiments are depicted. (**C**–**E**) Jurkat T-cells were transfected with either pEF-Tax-1, pEF-Tax-2, the listed plasmids encoding chimeras of both proteins, or the mock control vector pEF-1α (50 µg). (**C**) After 48 h, *Fascin* mRNA levels were measured by quantitative PCR (qPCR) and normalized to *β-actin* (ACTB). Resulting relative copy numbers (rcn) were normalized to untreated cells (UT), and the means of three independent experiments ± SD were compared using Student’s t test (* *p* < 0.05, ** *p* < 0.01, n.s., not significant). (**D**) Representative immunoblot of NF-κB2 (p100 and p52) and ACTB 48 h after transfection. Values depict densitometric analysis of p52 protein normalized to the house keeping gene HSP90 and the respective mock vector of the representative immunoblot. (**E**) Densitometric analysis of p100 protein normalized to ACTB is shown. Mean values of four independent experiments ± SE were compared using Student’s t test (* *p* < 0.05, ** *p* < 0.01). (**F**) Immunoblot of NF-κB2 (p100 and p52), Fascin, and ACTB of the HTLV-negative T-cell line Jurkat, one HTLV-1-positive (MT-2) and one Tax-1-transformed (Tesi) cell line, and two HTLV-2-positive cell lines (Mot, C3-44-Mo).

Compared to Tax-2, Tax-1 is a potent inducer of alternative NF-κB signaling [22,26]. To address the role of alternative NF-κB signaling in Fascin induction, we made use of Tax-1/Tax-2 chimeras that have been described to vary in their potential to activate alternative NF-κB signaling (Figure 5A) [26]. There are five aa in the leucine zipper region (aa 225-aa 232) of Tax-1 that differ from the aa present in the same region of Tax-2 and that are required for the full capacity of Tax-1 to increase alternative NF-κB signaling. In the used Tax-1/Tax-2 chimeras Tax 225-227, Tax 231-232, and Tax 225-232, regions of the Tax-1 protein are exchanged by those present in Tax-2 at the same positions. Tax300 consists of the first 300 N-terminal aa of Tax-2B and the C-terminal aa of Tax-1. To investigate the functionality of the Tax-1/Tax-2 chimeras, SVT35 reporter Jurkat T-cells carrying an NF-κB-dependent CD14 reporter were transfected with respective expression constructs. Flow cytometry of CD14 surface expression after 48 h post transfection revealed that Tax-1, all Tax-1/Tax-2 chimeras, and Tax-2B induced comparable amounts of CD14 expression (Figure 5B; ca. 54% of CD14 positive cells) compared to control cells that were transfected with an empty vector plasmid (pEF-1α; mock). This suggests that all Tax constructs activate classical NF-κB signals in T lymphocytes at equivalent amounts.

To test whether Tax-1/Tax-2 chimeras can induce *Fascin* transcription, normal Jurkat T-cells were transfected with the indicated constructs (Figure 5C). The increase in *Fascin* mRNA (Figure 5C) was different between the various Tax-chimeras. Tax-1 and Tax 225-227 enhanced *Fascin* mRNA expression to an equal amount, whereas Tax 231-232 induced *Fascin* mRNA only slightly (n.s.) more than Tax-1. Tax 225-232, however, increased *Fascin* mRNA slightly but not significantly less than Tax-1. Next, Tax 300 induced significantly less *Fascin* mRNA than Tax-1 (*p* < 0.05). Nevertheless, Tax 300 was still able to enhance *Fascin* transcript expression to a greater extent than Tax-2 (*p* < 0.05), which showed the lowest but still significantly increased *Fascin* copy numbers (compared to mock; *p* < 0.05) of all Tax expression constructs.

After identifying the varying potential of the Tax-1/Tax-2 chimeras to induce *Fascin* expression, we checked via immunoblot whether the proposed reduction of alternative NF-κB activation [26] could be confirmed and could be responsible for these differences (Figure 5D). As expected, Tax-1 induced the highest amount of p52 protein detected by densitometric analysis of the representative immunoblot (p52 protein normalized to mock; 11.9). The Tax-1/Tax-2 chimeras, however, displayed decreasing activity of alternative NF-κB signaling, whereby Tax 225-227 showed the highest amount of p52 (10.9), followed by Tax 231-232 (7.6), Tax 300 (4.7), and Tax 225-232 (3.8). This is in line with earlier studies showing that the Tax-1/Tax-2 chimera Tax 225-232 lacks a leucine zipper-like region crucial for activating the alternative NF-κB signaling pathway [26]. Once again, Tax-2 showed the lowest alternative NF-κB activity indicated by the lowest value of p52 protein expression (1.7). However, analysis of p100 expression revealed that all Tax constructs were able to significantly induce p100 expression compared to mock-transfected cells, and thus, classical NF-κB activity (Figure 5E; *p* < 0.05), confirming data obtained with the CD14 reporter (Figure 5B). In conclusion, we revealed a relation between alternative NF-κB signaling and Fascin expression: p100 processing to p52 is linked to transcriptional induction of *Fascin*.

Although HTLV-2 is not associated with leukemia in vivo, Tax-2 transforms T-cells in vitro. To examine whether Fascin is induced under conditions of Tax-2 transformation, we analyzed the expression of Fascin in HTLV-2-transformed T-cell lines. Immunoblots of two different HTLV-2-transformed T-cell lines, Mot and C3-44-Mo, were performed in comparison to the HTLV-negative cell line Jurkat, the HTLV-1-transformed cell line MT-2, and the Tax-1-transformed cell line Tesi (Figure 5F). In HTLV-1 and HTLV-2-positive cell lines, p100 expression was detectable and was the highest in the HTLV-2-transformed cell line Mot, reflecting the activity of the classical NF-κB signaling pathway. Contrary to Jurkat T-cells, p100 was largely processed to p52 in HTLV-1-, Tax-1-, and HTLV-2-transformed cell lines, confirming earlier observations in other cell lines [22,34]. The highest p52 expression was present in the Mot cell line, correlating with the elevated amount of p100 protein. We detected not only high amounts of Fascin protein in the HTLV-1- and Tax-1-transformed cell lines, but also comparable levels of Fascin protein in the two HTLV-2-transformed T-cell lines. Again, a link between Fascin induction and alternative NF-κB signaling was detected in HTLV-1- and HTLV-2-transformed cell lines. In conclusion, the sole expression of Tax-2 is not as efficient in amplifying alternative NF-κB signaling (as detected by p52 protein levels) and in inducing Fascin as the transformation of cells by HTLV-2 is. This suggests that either continuous expression of Tax-2 or other molecular factors of HTLV-2 transformation are involved in the activation of the alternative NF-κB signaling pathway, resulting in highly elevated Fascin expression. In summary, using Tax-1/Tax-2 chimeras as well as HTLV-2-transformed cell lines, we confirmed a link between the activity of the alternative NF-κB signaling pathway and the induction of Fascin in T-cells.

### 3.6. The SMAC-Mimetic AZD5582 Induces Alternative NF-κB Signaling but Not Fascin Expression

**Figure 6:**
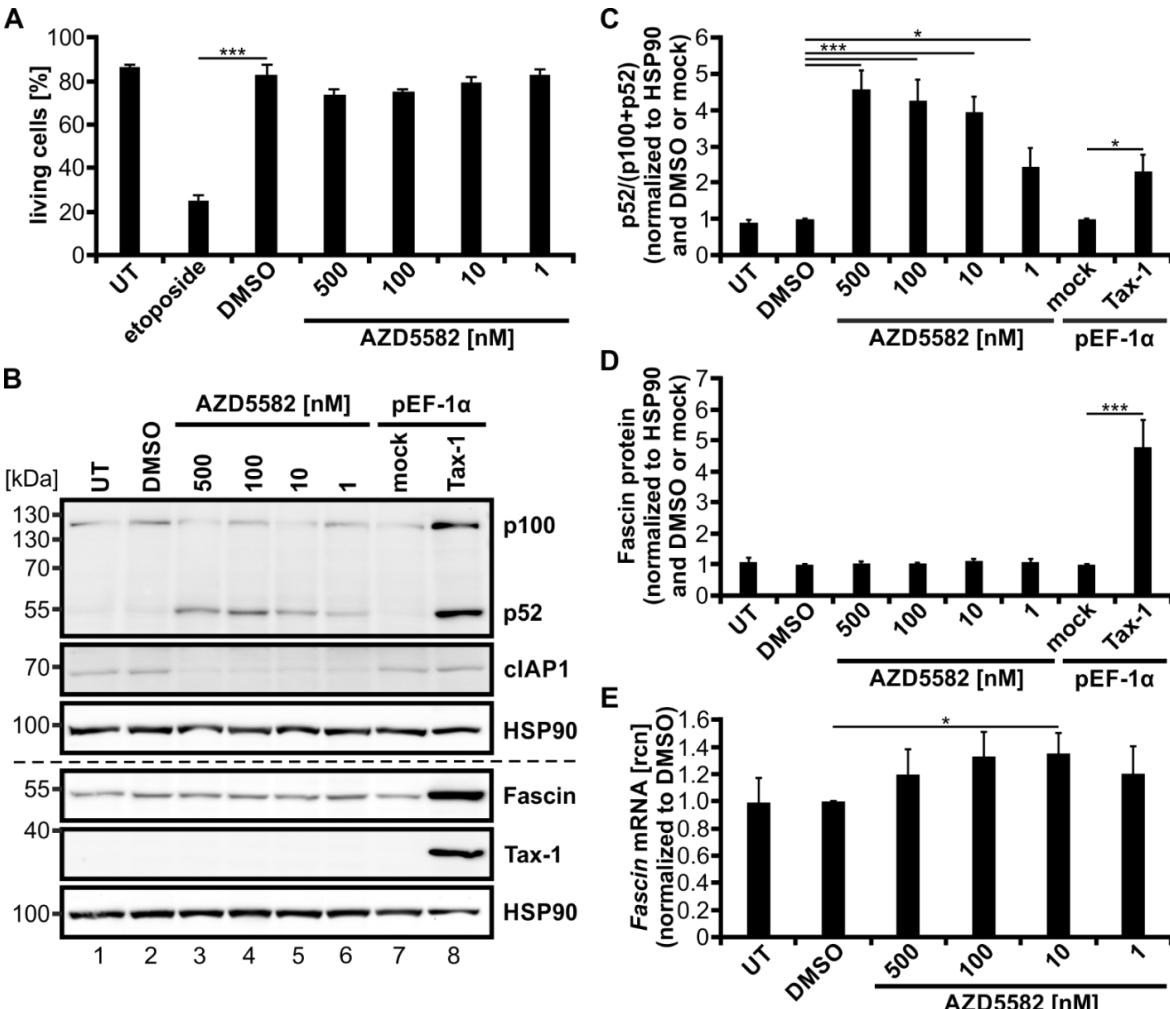
The second mitochondria-derived activator of caspases (SMAC)-mimetic AZD5582 induces alternative NF-κB activity but not Fascin expression. (**A**–**E**) Jurkat T-cells were treated for 48 h with different concentrations of AZD5582 (500, 100, 10, 1 nM) or dimethyl sulfoxide (DMSO) as a solvent control, or were left untreated (UT). (**A**) Cells were treated with 15 µM etoposide for 48 h as toxicity control. Vitality of the cells was estimated by propidium iodide staining followed by flow cytometry. The mean percentage of living cells of four independent experiments ± SE was compared using Student’s t test (*** *p* < 0.001). (**B**–**D**) Cells transfected with pEF-Tax-1 or the respective mock control vector pEF-1α (100 µg) served as positive or negative controls, respectively. (**B**) Representative immunoblot of NF-κB2 (p100 and p52), cellular inhibitor of apoptosis 1 (cIAP1), Fascin, Tax-1, and heat shock protein 90 (HSP90). (**C**,**D**) Densitometric analysis was performed to quantify p100, p52, and Fascin protein. Values were normalized to HSP90 and DMSO or pEF-1α, and the means of four independent experiments ± SE were compared using Student’s t test (* *p* < 0.05, *** *p* < 0.001). (**C**) To estimate p100 processing, p52 protein was divided by the total amount of p100 and p52 protein (p52/(p100 + p52)). (**E**) After 48 h, *Fascin* transcript levels were measured by quantitative PCR (qPCR) and normalized to *β-actin*. Resulting relative copy numbers (rcn) were normalized to those of DMSO-treated cells, and the means of four independent experiments ± SE were compared using Student’s t test (* *p* < 0.05).

To further elucidate its role in the regulation of Fascin expression, we strived to activate the alternative NF-κB signaling pathway independent of Tax-1. Therefore, the mimetic of the second mitochondria-derived activator of caspases (SMAC) AZD5582 was used, which is known to activate alternative NF-κB signaling in T-cells [61]. Jurkat T-cells were treated with different amounts of AZD5582 for 48 h, which had no toxic effects independent of the concentration used. In contrast, the positive control etoposide, a topoisomerase II inhibitor, significantly reduced cell vitality (Figure 6A, *p* < 0.001). The functionality of AZD5582 was confirmed by degradation of cIAP1 as well as processing of p100 to p52 via immunoblot (Figure 6B). Further, the processing of p100 to p52 was evaluated via densitometric analysis (Figure 6C; p52/(p100 + p52)) and showed a dose-dependent significant activation of alternative NF-κB signaling for all concentrations of AZD5582 used. Jurkat T-cells transfected with Tax-1 expression plasmids served as a positive control and indicated that not only p100 expression but also its processing into p52 was highly induced. The p52 levels once more coincided with a significantly elevated amount of Fascin protein (Figure 6B,D, *p* < 0.001), as described previously (Figure 1). Nevertheless, treatment of AZD5582 and the resulting activation of the alternative NF-κB pathway was insufficient to amplify Fascin on protein level (Figure 6B,D). On transcript level, however, little induction of *Fascin* was observed that was significant for one concentration of AZD5582 (Figure 6E; 10 nM, *p* < 0.05), but which by far did not reach the *Fascin* transcript levels elicited by Tax-1 dependent NF-κB signaling as demonstrated earlier (Figure 1). In conclusion, activation of the alternative NF-κB signaling pathway by treatment of Jurkat T-cells with the SMAC-mimetic AZD5582 is not sufficient in inducing strong and robust Fascin expression.

### 3.7. Activation of the Classical NF-κB Signaling by Tax-2 in Combination with Activation of Alternative NF-κB Signaling by the SMAC-Mimetic AZD5582 Induces Fascin Expression

**Figure 7:**
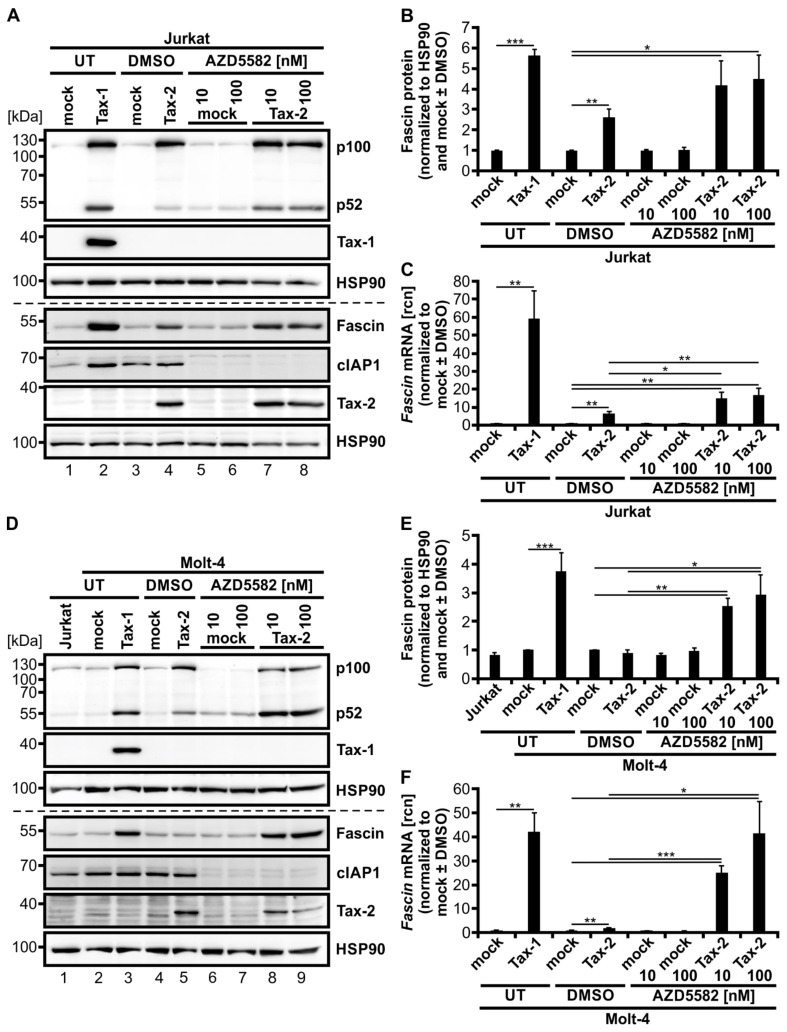
Activation of both classical and alternative NF-κB signaling by Tax-2 and the second mitochondria-derived activator of caspases (SMAC)-mimetic AZD5582 induces Fascin expression. (**A**–**C**) Jurkat T-cells or (**D**–**F**) Molt-4 T-cells were transfected with pEF-Tax-2, the mock control vector pEF-1α, or pEF-Tax-1 as positive control (100 µg). After 24 h, cells were treated with two concentrations of AZD5582 (10 or 100 nM) or dimethyl sulfoxide (DMSO) as solvent control for up to 48 h or were left untreated (UT). (**A**,**D**) Representative immunoblots of NF-κB2 (p100 and p52), Tax-1, Fascin, cellular inhibitor of apoptosis 1 (cIAP1), Tax-2, and heat shock protein 90 (HSP90). (**B**,**E**) Densitometric analysis was performed to quantify Fascin protein. Values were normalized to HSP90 and pEF-1α (mock) with or without DMSO as indicated and the means of (**B**) four or (**E**) three independent experiments ± SE were compared using Student’s t test (* *p* < 0.05, ** *p* < 0.01, *** *p* < 0.001). (**C**,**F**) *Fascin* transcript levels were measured by quantitative PCR (qPCR) and normalized to *β-actin*. Resulting relative copy numbers (rcn) were normalized to pEF-1α (mock) with or without DMSO as indicated and the means of (**C**) four or (**F**) three independent experiments ± SE were compared using Student’s t test (* *p* < 0.05, ** *p* < 0.01, *** *p* < 0.001).

**Figure A1:**
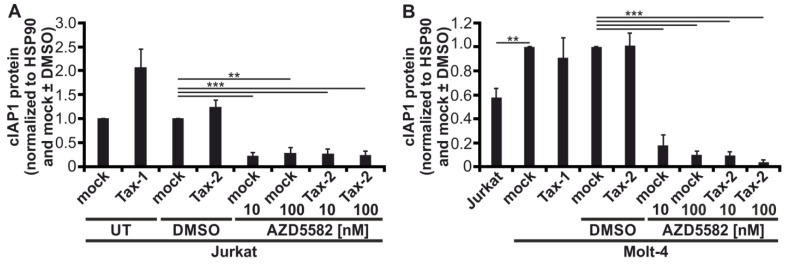
The second mitochondria-derived activator of caspases (SMAC)-mimetic AZD5582 leads to robust degradation of cellular inhibitor of apoptosis 1 (cIAP1). (**A**) Jurkat or (**B**) Molt-4 T-cells were transfected with pEF-Tax-2, the mock control vector pEF-1α, or pEF-Tax-1 as positive control (100 µg). After 24 h, cells were treated with two concentrations of AZD5582 (10 or 100 nM) or dimethyl sulfoxide (DMSO) as solvent control for up to 48 h or were left untreated (UT). Immunoblots are shown in Figure 7A (Jurkat) and Figure 7D (Molt-4). Densitometric analysis was performed to quantify cIAP1 protein. Values were normalized to HSP90 and pEF-1α (mock) with or without DMSO as indicated, and the means of (**A**) four or (**B**) three independent experiments ± SE were compared using Student’s t test (** *p* < 0.01, *** *p* < 0.001).

Finally, we wanted to test if combining induction of the alternative NF-κB signaling pathway by AZD5582 with activating classical NF-κB signaling by Tax-2 results in a strong induction of Fascin and can ultimately mimic its regulation by Tax-1, which highly activates both pathways in Jurkat T-cells. Therefore, Jurkat T-cells were transfected with Tax-2 or the mock vector pEF-1α and, after 24 h, treated with 10 or 100 nM AZD5582 for up to 48 h (Figure 7). Tax-1-transfected cells once more served as a positive control for simultaneous activation of classical and alternative NF-κB signaling, resulting in highly elevated Fascin expression as confirmed by immunoblot (Figure 7A,B) and qPCR (Figure 7C). All cells treated with AZD5582 showed degradation of cIAP1 by approximately 75% (Figure 7A and Figure A1A). Levels of p52 protein were similar after AZD5582 treatment alone and after transfection with Tax-2, but induction of p100 was absent comparable to DMSO-treated and untreated mock-transfected samples. In contrast to Tax-2 (Figure 7B,C; *p* < 0.01), both concentrations of AZD5582 were again insufficient to elevate Fascin on protein (Figure 7B) and transcript levels (Figure 7C) in this setting. However, the combination of AZD5582 treatment with Tax-2 transfection ultimately induced p52 and both Fascin protein (Figure 7B; *p* < 0.05) and transcript expression (Figure 7C, *p* < 0.01) in Jurkat T-cells.

**Figure A2:**
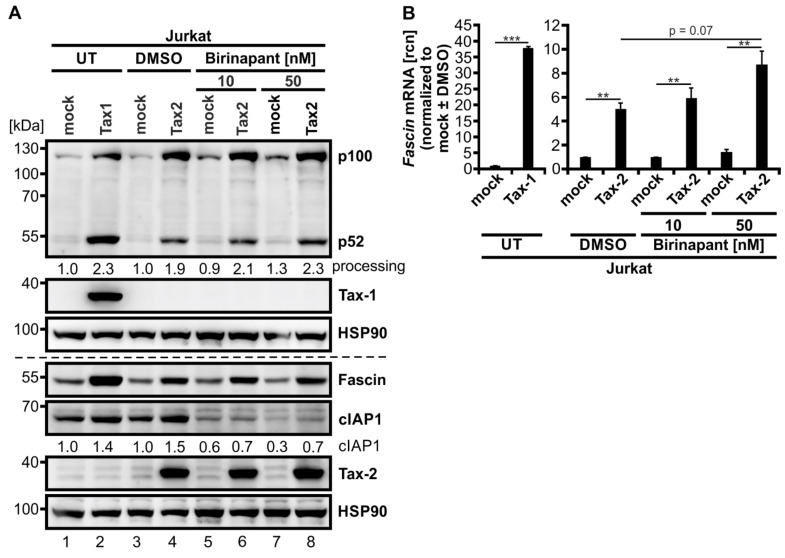
Impact of the SMAC-mimetic Birinapant on cIAP1 degradation, p100 processing and Fascin induction. (**A**,**B**) Jurkat T-cells were transfected with pEF-Tax-2, the mock control vector pEF-1α, or pEF-Tax-1 as positive control (100 µg). After 24 h, cells were treated with two concentrations of Birinapant (10 nM, 50 nM) or dimethyl sulfoxide (DMSO) as solvent control for 24 h or were left untreated (UT). (**A**) Representative immunoblots of NF-κB2 (p100 and p52), Tax-1, Fascin, cellular inhibitor of apoptosis 1 (cIAP1), Tax-2, and heat shock protein 90 (HSP90). Densitometric analysis was performed to quantify cIAP1 protein. Values were normalized to HSP90 and pEF-1α (mock) with or without DMSO. To estimate p100 processing, p52 protein was divided by the total amount of p100 and p52 protein (p52/(p100 + p52)). (**B**) *Fascin* transcript levels were measured by quantitative PCR (qPCR) and normalized to *β-actin*. Resulting relative copy numbers (rcn) were normalized to pEF-1α (mock) with or without DMSO as indicated and the means of three independent experiments ± SE were compared using Student’s t test (** *p* < 0.01; *** *p* < 0.001). UT, untreated cells.

To further enlighten the combinatory effects of classical and alternative NF-κB signaling on Fascin regulation in T-cells, we performed the same experiment in a second CD4^+^ T-cell line, Molt-4. As earlier results indicated (Figure 1E,F), Tax-2 alone was insufficient in significantly inducing Fascin protein expression despite robust activation of both classical and alternative NF-κB signaling in Molt-4 T-cells, which is reflected by elevated p100 and p52 protein levels (Figure 7D,E). Only on the transcript level, Tax-2 alone lead to a significant *Fascin* induction (Figure 7F; *p* < 0.01), but it was less than that observed in Jurkat T-cells (Figure 7C). Treatment with AZD5582 enhanced cIAP1 degradation by approximately 90% (Figure 7D and Figure A1B) and p52 protein independent of p100 induction (Figure 7D), but Fascin protein (Figure 7E) or mRNA levels (Figure 7F) were unaffected in Molt-4 T-cells as well. After combining AZD5582 treatment with Tax-2 expression, not only Fascin protein (Figure 7E; *p* < 0.05) but also *Fascin* transcripts (Figure 7F; *p* < 0.05) were significantly elevated by the combinatory activation of classical and alternative NF-κB signaling, both compared to mock and to Tax-2-transfected cells. Different than in Jurkat T-cells, the combinatory *Fascin* transcript induction with the highest concentration of AZD5582 used (100 nM) reached comparable levels as those observed by Tax-1-dependent induction in Molt-4 T-cells (Figure 7F). Use of the SMAC-mimetic Birinapant, which has been described to activate alternative NF-κB activity in multiple myeloma cells [62], revealed that this compound is less active than AZD5582 in degrading cIAP1 in the Jurkat T-cell line (see Figure A2A; cIAP1 expression diminished by approximately 40%), and also less effective in selectively inducing processing of p100 into p52 in Jurkat T-cells (Figure A2A). Consequently, the impact of Birinapant on enhancing Tax-2-mediated induction of *Fascin* transcription was visible but moderate (Figure A2B; *p* = 0.07). Therefore, we conclude that AZD5582 is superior to Birinapant in inducing alternative NF-κB activity, and thus *Fascin* transcription, in Jurkat T-cells. In summary, we were able to recreate Tax-1-like effects on Fascin regulation in T-cells by combining the related oncoprotein Tax-2 with the SMAC-mimetic AZD5582, resulting in both strong classical and alternative NF-κB activity and, thus, in Fascin induction. This finally demonstrates the necessity of the activity of both the classical and alternative NF-κB signaling pathways at the same time to elicit a strong and robust Fascin induction in T-cells, making Tax-1 and Tax-2 useful tools to study oncogenic signaling in T-cells.

## 4. Discussion

The actin-bundling protein Fascin is upregulated in many types of cancer, including virus-induced cancers such as ATLL [36], a neoplasia caused by the oncogenic retrovirus HTLV-1. Functionally, Fascin contributes to the migration and invasion of cancer cells and is thereby crucial for stability and formation of specialized protrusive structures, so-called invadopodia [63]. Transcriptional regulation of Fascin is heterogeneous between different cell types, tissues, and tumors [63,64]. We have previously shown that a single viral oncoprotein, Tax-1 of HTLV-1, is a potent inducer of Fascin in T-cells depending on classical NF-κB signaling [36,41]. In this study, we compared Tax-1 with Tax-2 from the closely related but non-oncogenic HTLV-2 to investigate the regulation of Fascin expression in T-cells. We found that transcriptional activation of Fascin by these viral oncoproteins depends on activity of both the classical and the alternative NF-κB signaling cascade. In contrast to earlier findings [22,26], our study also shows that Tax-2 is able to induce alternative NF-κB signaling in two different CD4^+^ T-cell lines, albeit at lower levels than Tax-1. Together, Tax-1 and Tax-2 proteins are useful tools to study oncogenic signaling in T-cells.

Fascin induction by Tax-1 depends on classical NF-κB signaling [36]; however, since blocking of the classical pathway also leads to inhibition of alternative NF-κB signaling due to the crosstalk between both NF-κB pathways [65,66,67], a contribution of the alternative NF-κB pathway to Fascin regulation could not be fully excluded. Therefore, we sought to compare Tax-1 from HTLV-1, a potent inducer of classical and alternative NF-κB signaling [29,68], to Tax-2 from the non-oncogenic HTLV-2B, which has been described to activate classical but not alternative NF-κB signaling [22,26]. We found that not only Tax-1 but also Tax-2B is able to activate *Fascin* expression upon transient transfection in Jurkat and Molt-4 T-cells, albeit at a much lower level than Tax-1 and with slight differences in the two T-cell lines: while Tax-2 led to induction of both *Fascin* mRNA and protein in Jurkat T-cells, only *Fascin* mRNA was induced in Molt-4 T-cells, which may be due to differences between the cell lines or the transcriptional induction of the endogenous *Fascin* promoter. Expression of Fascin could be linked to the activity of the alternative NF-κB signaling pathway, which is reflected by processing of the NF-κB2 precursor p100 into p52. To our surprise, we found that not only Tax-1 but also Tax-2 was able to induce processing of p100 into p52 in Jurkat and Molt-4 T-cells. To strengthen our findings, we made use of two different Tax-2B expression constructs. Originally, the *tax-2B* coding sequence isolated from the Italian patient PR-46 by Turci, Romanelli, Lorenzi, Righi and Bertazzoni [57] was used to identify the NLD present at the N-terminal region of Tax-2. This sequence was later cloned into the pcDNA6.2 expression vector, and the internal FLAG-6His tag was inserted at position 337, resulting in the pcTax-2F construct [57]. The coding sequence of the isolate from a North American intravenous drug user cloned into pEF-Tax-2 was shown to be able to induce classical NF-κB and CREB signaling comparable to other Tax subtypes [26,58]. This is in line with our observations since expression of both constructs (pEF-Tax-2, pcTax-2F) not only strongly induced p100 expression, a target of the classical NF-κB signaling pathway (Figure 1B), but expression of both constructs also led to the processing of p100 into p52, which reflects activity of the alternative NF-κB pathway. Thus, neither the differences in the coding sequence nor in the integration of the internal FLAG-6His tag in pc-Tax-2F lead to alterations in classical and alternative NF-κB activation and induction of Fascin expression by the two Tax-2B constructs.

Transcriptional regulation of *Fascin* can take place at different levels involving the promoter, silencer or enhancer elements, chromatin, or the 3′ untranslated region (3′ UTR) as well as microRNAs [40,50]. In this study, we found that both Tax-1 and Tax-2 transactivate a fragment of the human *Fascin* promoter at comparable levels, depending on classical NF-κB signaling. Earlier analysis of the human *Fascin* promoter performed with reporter plasmids with progressive 5′ deletions identified a core promoter that showed basal activity in Jurkat T-cells (−88/+123) [41] and has already been described in dendritic cells (DCs) [50]. Additionally, a 1.6 kb fragment of the *Fascin* promoter (phF1.6) was discovered that is responsive to Tax-1 [41] and, as shown here, is similar to Tax-2. The Tax-1-specific effect on activation of the *Fascin* promoter was lost in 5′ deleted promoter constructs shorter than 1.4 kb and in promoter constructs longer than 1.6 kb [41], suggesting that this Tax-responsive promoter region (TRR; −1499/−1325) is surrounded by repressor elements, a fact that could also be demonstrated in DCs [50]. Activation of phF1.6 by Tax-1 and Tax-2 could be attributed to classical NF-κB signaling: (1) inhibition of IKK2 by a chemical compound [69,70], (2) inhibition of IκB by co-transfection of a dominant-negative inhibitor [54], and, in case of Tax-1, (3) NF-κB-deficient Tax-mutants [71], led to decreased Tax-mediated transactivation of the *Fascin* promotor ([41]; this work), which is in line with earlier observations in human breast cancer cell lines [72,73]. However, sole stimulation with strong chemical inducers of the classical NF-κB pathway, tumor necrosis factor (TNF) and 12-O-Tetradecanoylphorbol-13-acetate (TPA), was neither sufficient to activate the *Fascin* promoter in Jurkat T-cells nor to induce *Fascin* transcripts [41]. These findings suggest that Tax-1 and Tax-2 (1) activate the NF-κB pathway in a different manner than TPA and TNF-α, (2) induce another signaling pathway apart from NF-κB which is crucial for proper *Fascin* transactivation, or (3) regulate Fascin expression indirectly by NF-κB-dependent target genes. One potential candidate could be the TNFR superfamily member CD40, which is regulated by Tax-1 dependent on NF-κB [74], and plays a role in inducing *Fascin* expression in dendritic cells [75]. It is unlikely that alternative NF-κB signaling plays a role in activating the *Fascin* promoter since Tax-2 and Tax-1 did not differ in their capacity to activate the human *Fascin* promoter phF1.6, while Tax-2 is only a weak while Tax-1 is a strong inducer of alternative NF-κB activity, as shown in this work. A closer look at the isolated TRR cloned in front of the core promoter revealed that this region can still be activated by Tax-1 and Tax-2, albeit to a lesser extent than the full length phF1.6, identifying the TRR as an element potentially contributing to enhancer functions. However, the TRR does not contain any putative NF-κB binding sites [41]. As discussed earlier [41], putative cAMP response element-binding protein (CREB) responsive regions could be localized within the TRR and the core promoter, which were identified as Tax-response elements (TRE). The TRE is a 21 bp triple repeat, containing an octamer motif TGACG(T/A)(C/G)(T/A) homologous to the cAMP response element 5′-TGACGTCA-3′ that is flanked by a GC rich region [76,77]. Tax-1 is not able to bind directly to the TRE DNA [78,79], and there is no experimental evidence that Tax proteins bind to the TRR of the human *Fascin* promoter. Nevertheless, the interaction of Tax-1 with phosphorylated CREB/activating transcription factors (ATF), particularly with the basic leucine zipper domain [80], leads to the formation of a ternary complex with the TRE [60,81,82,83]. Together, since Tax-1 and Tax-2 did not differ significantly in inducing activation of the *Fascin* promoter constructs, this suggests that the identified phF1.6 and the TRR could contribute to transcriptional regulation of Fascin upon expression of Tax proteins, but they are not pivotal for full activation of *Fascin* mRNA production.

Treatment of cells with actinomycin D confirmed that Tax-1 is superior in inducing Fascin expression to Tax-2; however, these experiments also revealed that stability of *Fascin* mRNA does not differ between Tax-1- and Tax-2-expressing cells, suggesting that post-transcriptional regulation of Fascin is unaffected by the Tax proteins. It could still be possible that regulation of Fascin occurs via distant control elements, e.g., in *Fascin* introns or enhancer elements. This is supported by earlier findings of our group, which showed that Tax-1-mediated Fascin induction also depends on a promoter-independent pathway that is sensitive to the Src kinase inhibitor PP2 [41]. Yet, it is unclear whether PP2 also impacts Tax-2 mediated regulation of Fascin.

Despite a sequence homology of about 85%, there are major differences between Tax-1 and Tax-2 [68,84,85,86]. While Tax-1 harbors a C-terminal PBM, Tax-2 is devoid of a PBM [21,87]. Investigation of the Tax-1 deletion mutant TD319 [51], which lacks the PBM, revealed that the PBM is necessary for full transcriptional induction of *Fascin* and thus contributes to Fascin regulation. This is in line with earlier work, which has shown that Tax-1 lacking the PBM is slightly impaired in alternative NF-κB signaling as reflected by diminished p100 processing [22] and in full activation of an NF-κB-dependent reporter [25]. Additionally, the PBM is responsible for HTLV-1-induced primary T-cell proliferation and is involved in the induction of micronuclei [25]. Further, the interaction of Tax-1 with PDZ-containing proteins might play a role in Tax-1 transforming activity [21,23,25]. Moreover, PBMs have been detected in other oncogenic viruses, including human papillomavirus and adenovirus, proposing a role of PBM and PDZ-containing proteins in cellular transformation [22,88].

Another crucial difference between Tax-1 and Tax-2 is determined by a leucine-rich region in Tax-1 (aa 225-232), which is missing in Tax-2 but is important for Tax-1-induced p100 processing [26]. Making use of Tax-1-/Tax-2-chimeras, we confirmed that chimeras lacking aa 225-232 are not only impaired in alternative NF-κB signaling [26] but also in the induction of Fascin. When comparing several Tax-1/Tax-2 chimeras with Tax-1 and Tax-2, we found a link between the activity of the alternative NF-κB signaling pathway and induction of Fascin expression. While earlier work argues that induction of the alternative NF-κB pathway is specific to Tax-1 [26,89] since Tax-2 does not interact with p100 [22], we see clear alternative NF-κB activity upon overexpression of Tax-2 in two different CD4^+^ T-cell lines in this study as reflected by the processing of p100 into p52, albeit at much lower levels than observed with Tax-1. Other groups were also previously able to observe a limited but unneglectable p100 processing after stable expression of Tax-2, even though they did not further discuss or quantify it; thus, no statistical evaluation was performed [26,90]. Hence, in our study, we conducted densitometric analysis to quantify p52 protein levels or p100 processing, which clearly demonstrated activity of alternative NF-κB signaling. Conclusively, not only our work but also previous studies made clear that Tax-2 is far less potent in inducing alternative NF-κB signaling than Tax-1, and its minimal impact probably plays a limited role in the induction of this pathway in natural infections. Since HTLV-2 is predominantly found in CD8^+^ T-cells in infected patients, it remains to be determined whether Tax-2 has a similar impact on alternative NF-κB activity in CD8^+^ T-cells such as in CD4^+^ T-cells, as described in this study. However, we and others were able to observe strong processing of p100, implying activity of the alternative NF-κB pathway not only in HTLV-1- or Tax-1-transformed but also in HTLV-2-transformed cell lines (Figure 5F, [22]). In these cells, the viral Tax oncoproteins are continuously expressed and unfold their in vitro transforming potential, which has a possible role in the induction of alternative NF-κB signaling. However, because of its limitations discussed earlier, Tax-2 is probably not the only factor necessary for induction of the alternative NF-κB pathway during HTLV-2 in vitro transformation. It might be possible that continuous expression of Tax-2 also exhibits properties to impair genetic stability comparable to Tax-1. This could explain the high activity of alternative NF-κB signaling, as measured by the high processing rates of p100 into p52, that is predominantly visible after in vitro transformation of cells by HTLV-2 (Figure 5E; [22]), but only moderately after transient expression of Tax-2. Therefore, other determining factors need to be investigated in the future. Apart from the viral oncoprotein Tax-1, latent membrane protein 1 (LMP-1) from Epstein–Barr virus, viral Fas-associated death domain-like IL-1-converting enzyme inhibitory protein (v-FLIP)/K13 from Kaposi’s sarcoma-associated herpesvirus (KSHV), or the Tio oncoprotein of Herpesvirus ateles are potent inducers of the alternative NF-κB pathway [91,92,93,94]. While we recently showed that LMP-1 is also a potent inducer of Fascin [55], this remains to be determined for the other viral oncoproteins.

The relevance of alternative NF-κB signaling for oncoprotein-dependent regulation of Fascin was also strengthened by our findings using the SMAC-mimetic AZD5582. While AZD5582 led to robust induction of alternative NF-κB signaling confirming earlier observations [61], Fascin protein expression was not at all, and *Fascin* transcripts were only slightly affected by the compound. However, by co-administration of AZD5582 together with Tax-2, we were able to recreate Tax-1-like effects on Fascin regulation in T-cells, resulting in both strong classical and alternative NF-κB activity and thus in enhanced Fascin induction. In contrast, another SMAC-mimetic, Birinapant [62], was less effective in cIAP1 degradation, p52 induction, and thus Fascin induction, supporting the notion that AZD5582 is a more potent activator of alternative NF-κB activity than Birinapant in Jurkat T-cells.

Taken together, this work sheds new light on the transcriptional regulation of the tumor marker Fascin by oncoproteins and identifies a cooperative effect of classical and alternative NF-κB activity to be crucial for induction of Fascin expression in CD4^+^ T-cells. Not only in the context of HTLV, but also in other disorders, such as inflammatory bowel disease, alternative NF-κB is aberrantly deregulated and is associated with poor response to therapy [95]. However, in contrast to the classical NF-κB pathway, the clinical significance of alternative NF-κB signaling is less well understood. With our study, we were able to enlighten the regulation of alternative NF-κB in HTLV-1 and HTLV-2 infections and identified Tax-1 and Tax-2 to be useful tools in studying oncogenic signaling.

## Data Availability

Data are contained within the article and Supplementary Material.

## Supplementary Materials

The following are available online at https://www.mdpi.com/article/10.3390/cancers14030537/s1, Original images of immunoblots.
