# Supplementary material for: Alternative NF-κB Signaling Discriminates Induction of the Tumor Marker Fascin by the Viral Oncoproteins Tax-1 and Tax-2 of Human T-Cell Leukemia Viruses
Source: Cancers (Basel). 2022 Jan 21;14(3):537. doi: 10.3390/cancers14030537 (PMC8833421; doi:10.3390/cancers14030537)
Supplement: Supplementary file 1 [file cancers-14-00537-s001.zip › cancers-1491095-supplementary.pdf]

Supplementary Data

Heym et al.

Photographs of Western blots detected by chemiluminescence and a separate photograph of the marker are shown (left side). The photograph of the marker was taken with the same camera and with the same position of the blot (but without chemiluminescence) and was overlaid with the same lane of the chemiluminescent photograph

Figure 1B   Heym et al.

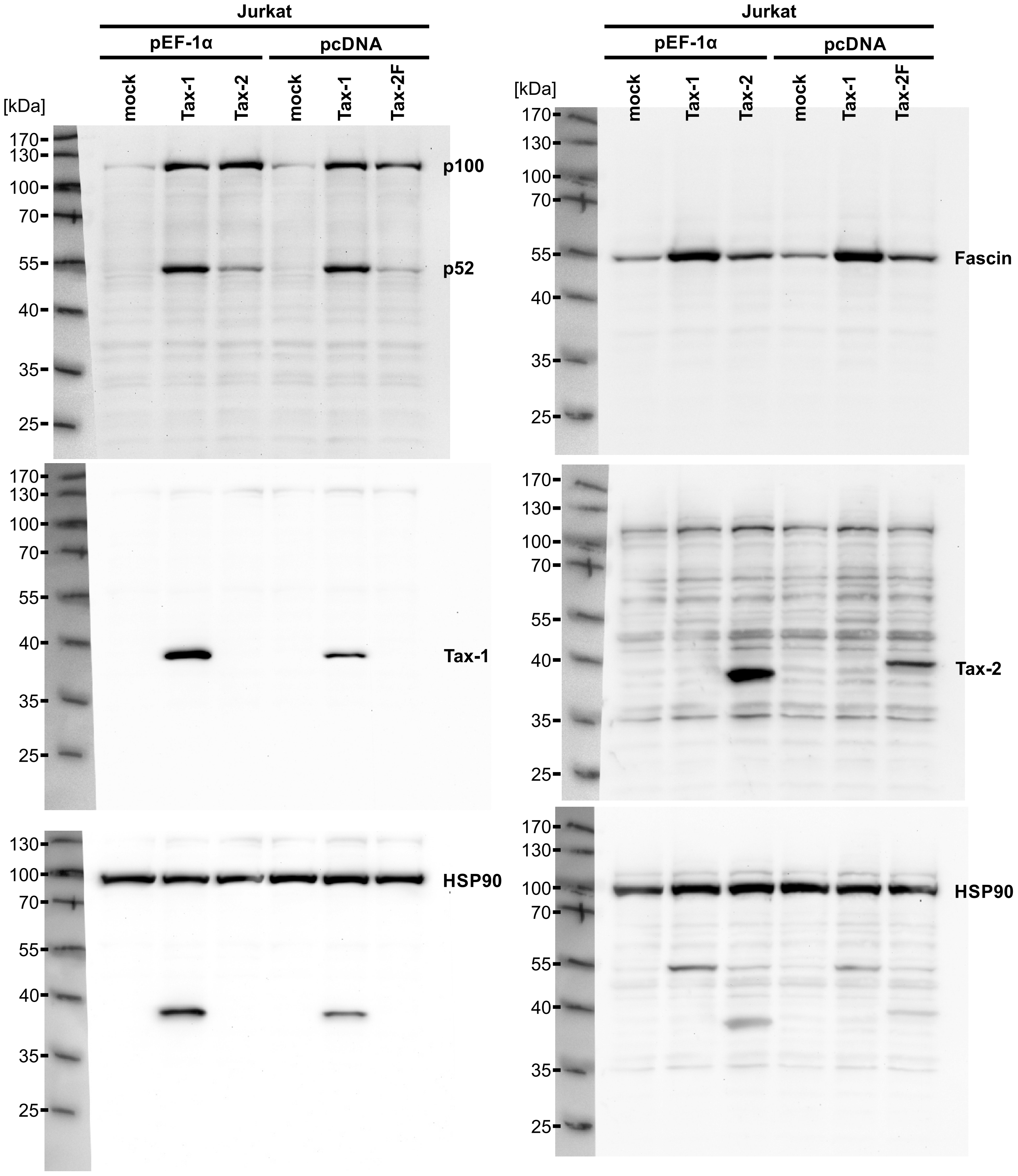

Ratio normalized to HSP90	pEF-1α			pcDNA		
	mock	Tax-1	Tax-2	mock	Tax-1	Tax-2
**p100**	0.08	0.81	1.05	0.14	0.73	0.56
**p52**	0.08	0.99	0.42	0.07	0.81	0.20
**Tax-1**	0.00	1.30	0.00	0.00	0.52	0.00
**Fascin**	0.41	0.97	0.53	0.28	1.13	0.63
**Tax-2**	0.02	0.06	0.97	0.08	0.14	0.69

Figure 1E Heym et al.

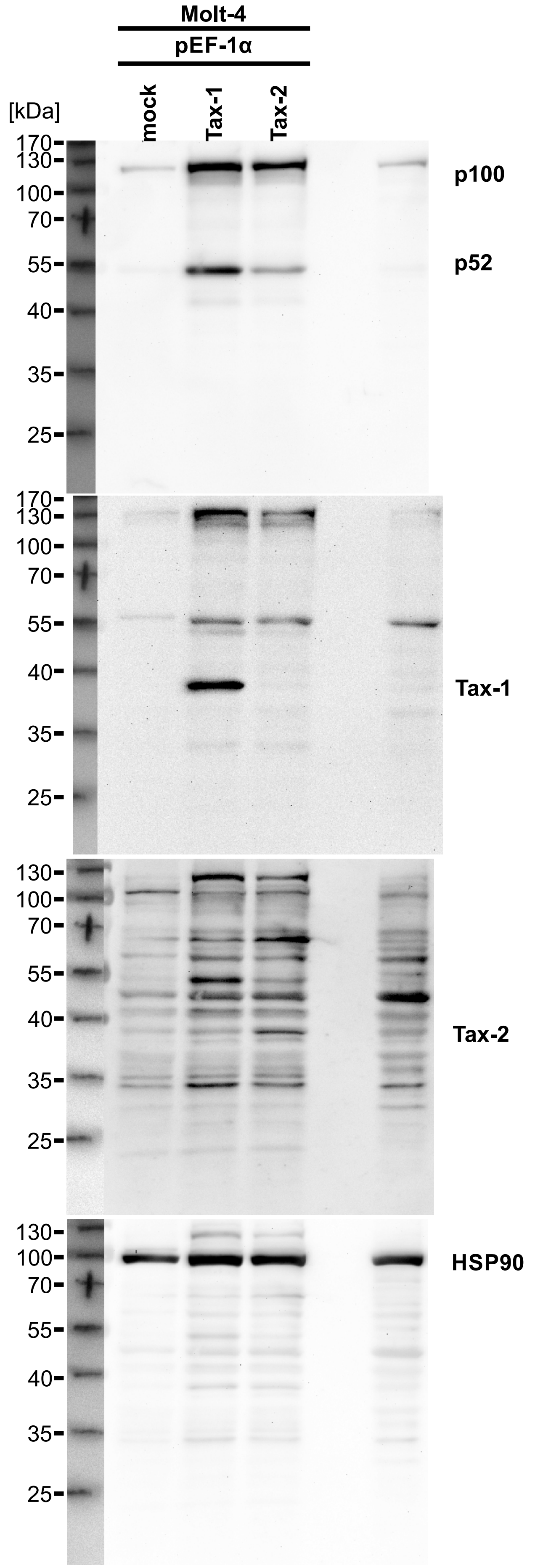

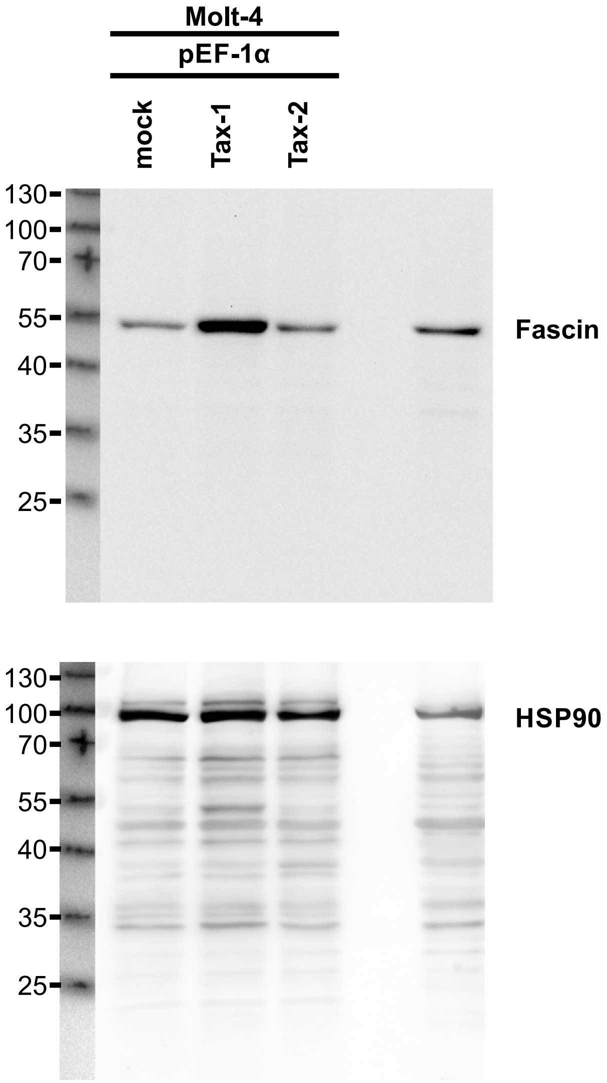

Ratio normalized to HSP90	pEF-1α		
	mock	Tax-1	Tax-2
**p100**	0.15	0.96	0.88
**p52**	0.03	0.71	0.28
**Tax-1**	0.02	1.00	0.06
**Tax-2**	0.15	0.24	0.62
**Fascin**	0.30	1.04	0.42

Figure 2B Heym et al.

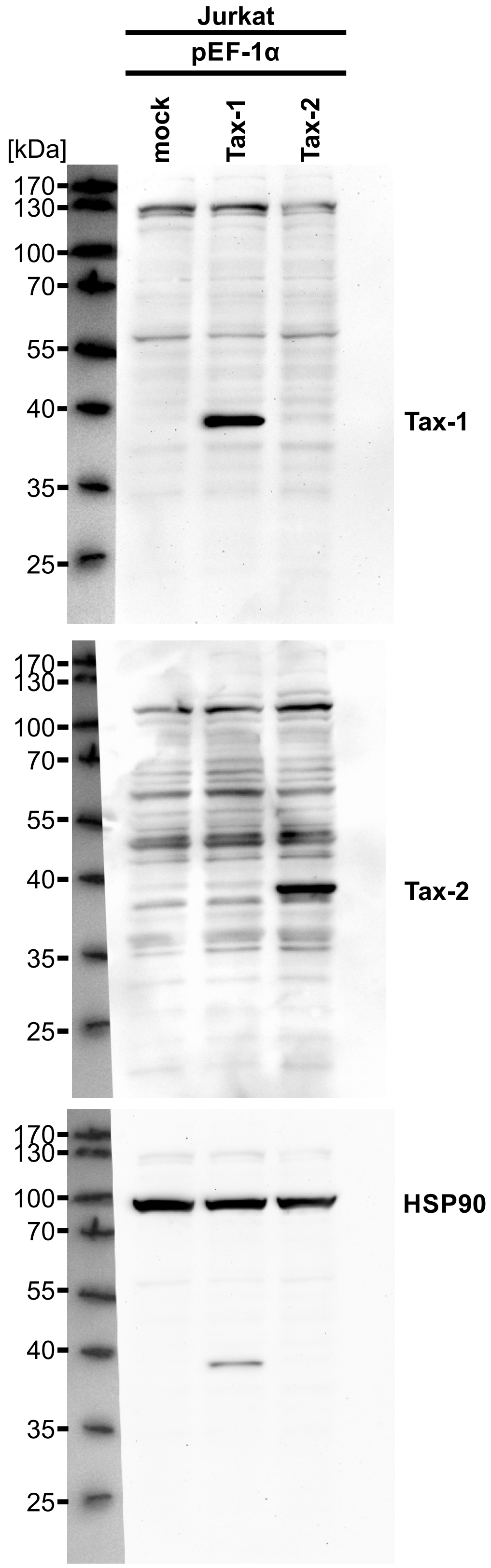

Ratio normalized to HSP90	Jurkat		
	phF-1.6		
	mock	Tax-1	Tax-2
Tax-1	0.00	1.09	0.04
Tax-2	0.00	0.08	1.31

Fig. 2C Fig. 2G

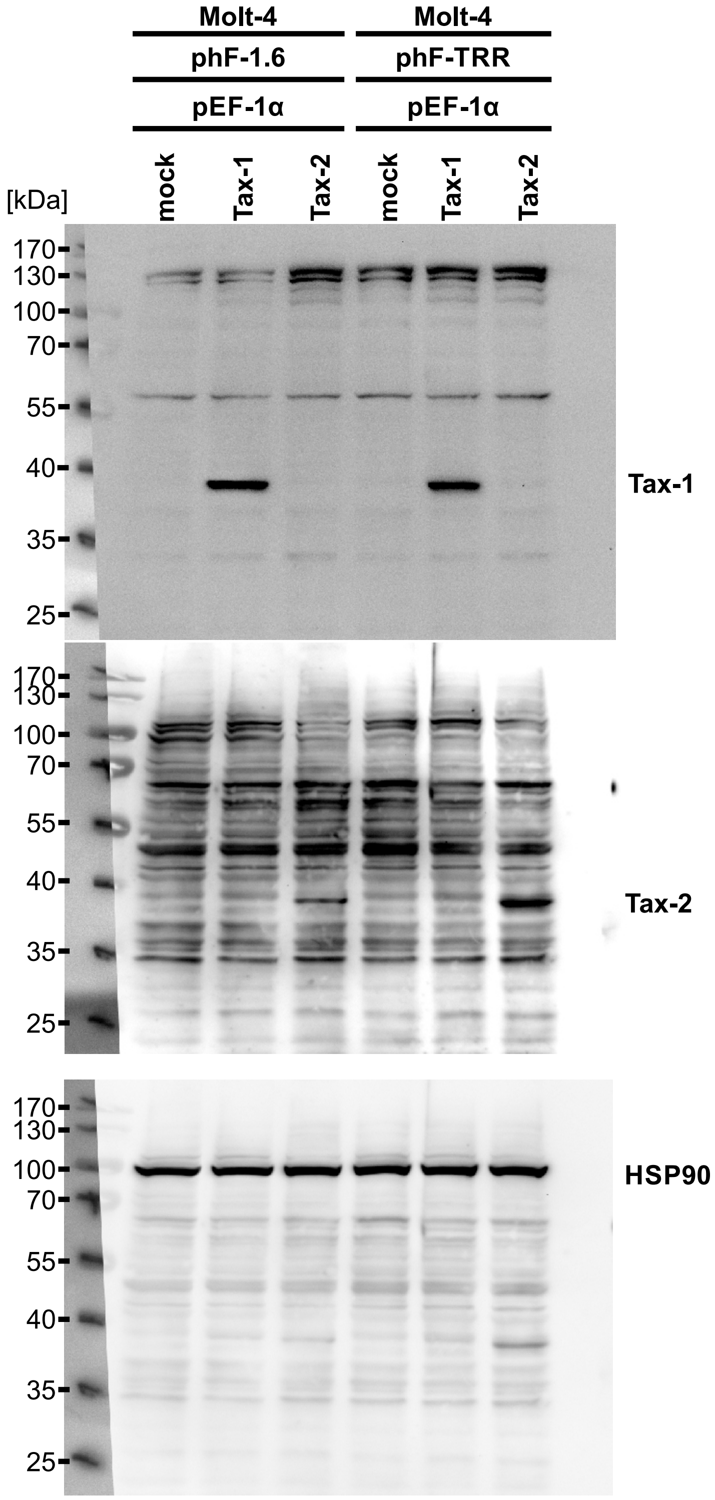

Ratio normalized to HSP90	Molt-4					
	phF-1.6			phF-TRR		
	mock	Tax-1	Tax-2	mock	Tax-1	Tax-2
Tax-1	0.01	1.09	0.04	0.04	0.88	0.03
Tax-2	0.30	0.23	0.72	0.23	0.27	1.18

Figure 2F Heym et al.

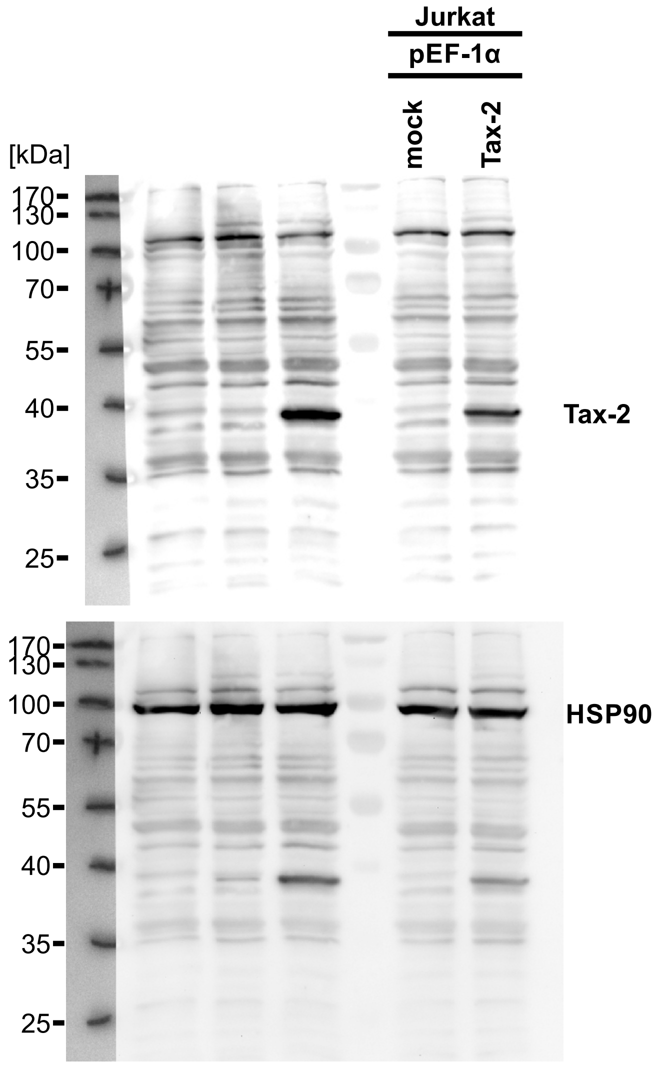

Ratio normalized to HSP90	Jurkat	
	phF-TRR	
	mock	Tax-2
**Tax-2**	0.1	1.2

Figure 4 Heym et al.

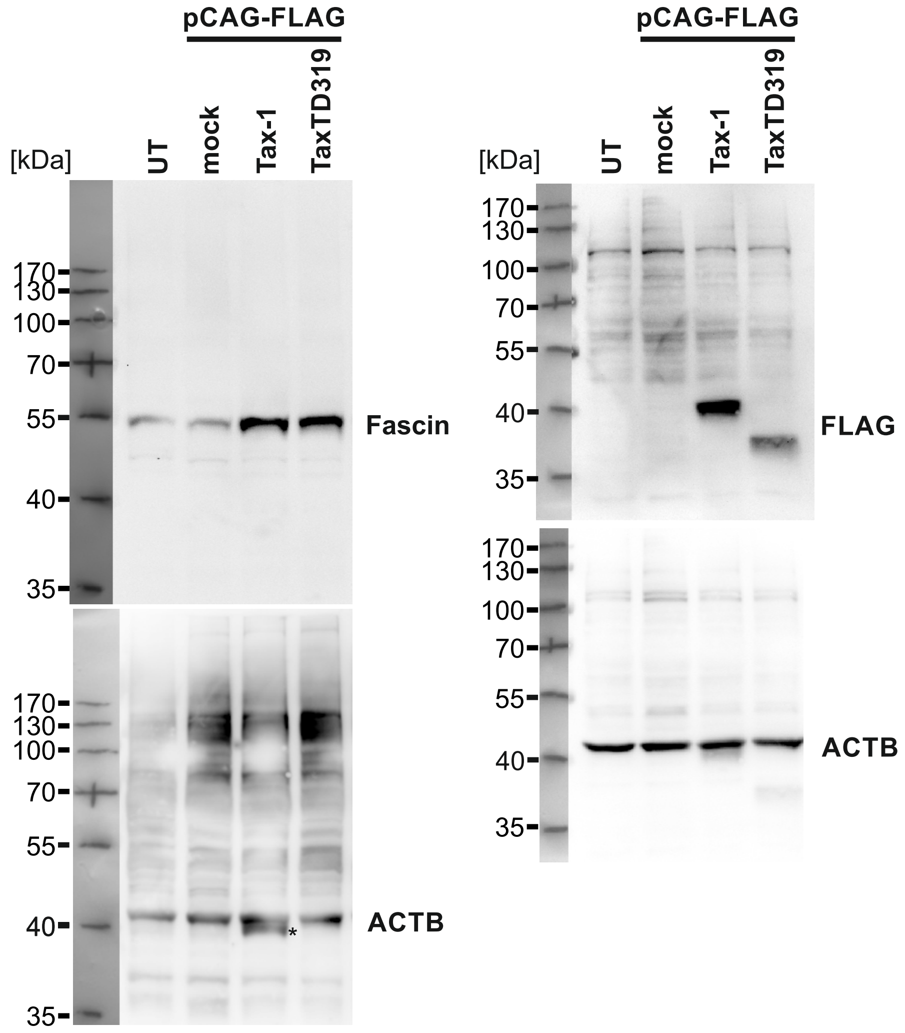

*indicates unrelated staining

Ratio normalized to ACTB	UT	pCAG-FLAG		
		**mock**	**Tax-1**	**TaxTD319**
**Fascin**	0.54	0.45	1.28	1.41
**FLAG**	0.03	0.20	1.17	0.85

Figure 5 Heym et al.

D

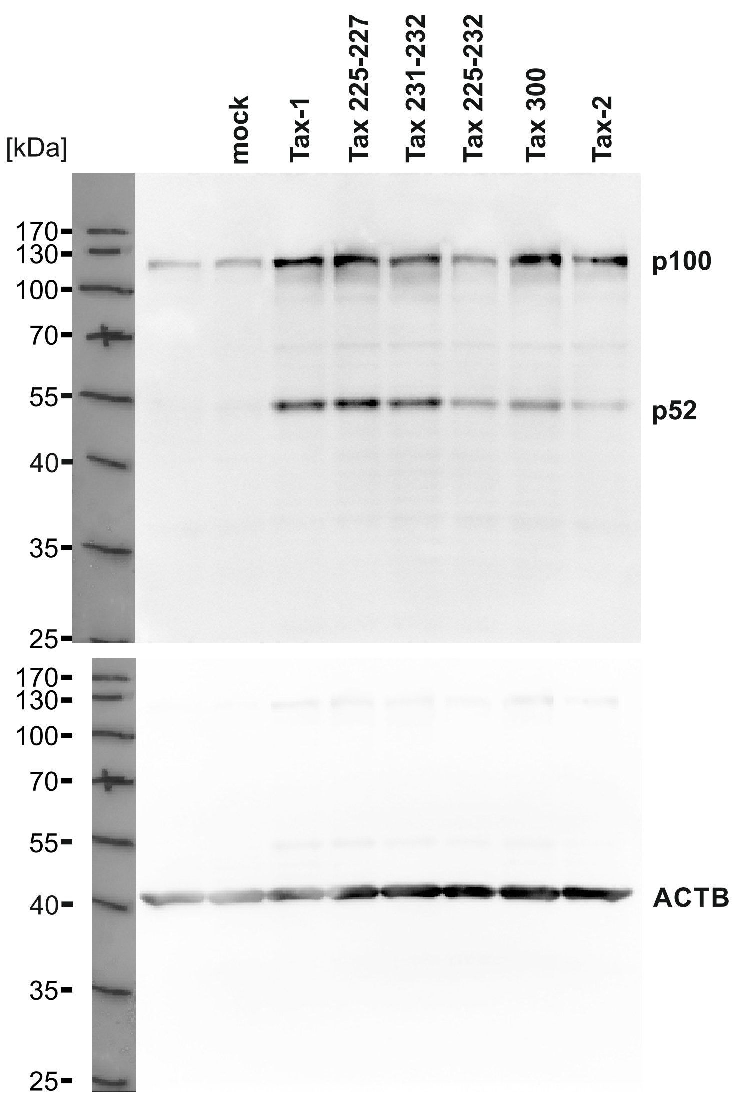

E

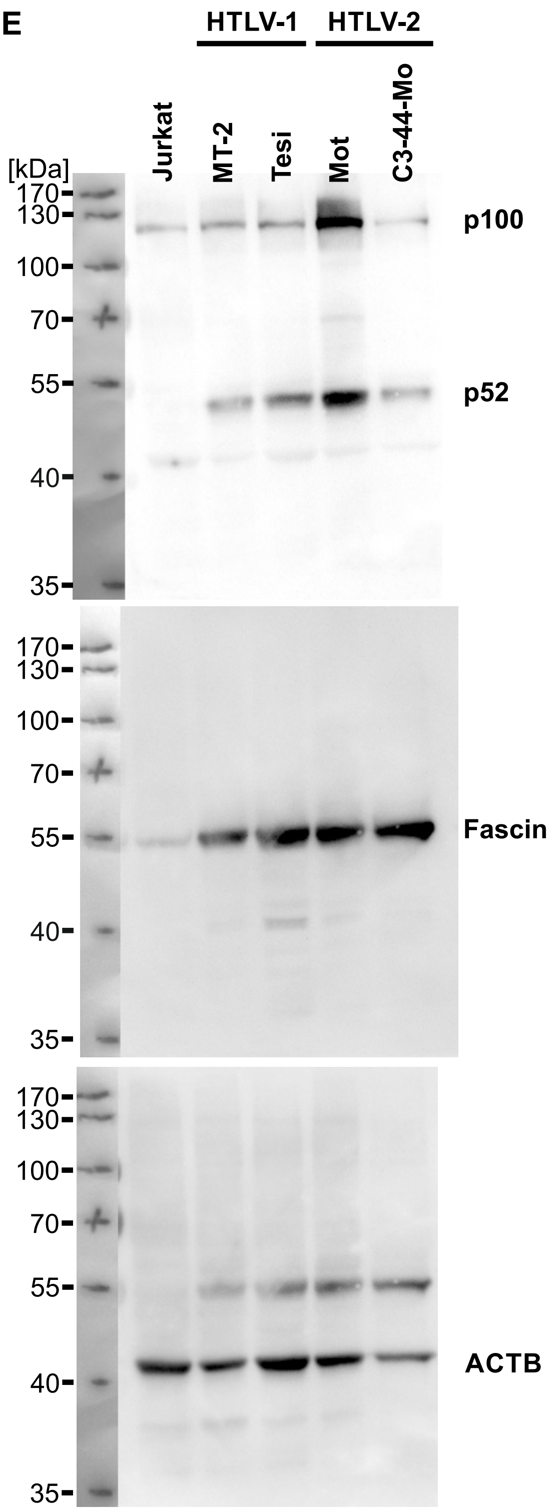

Ratio (normalized to ACTB)	mock	Tax-1	Tax 225-227	Tax 231-232	Tax 225-232	Tax 300	Tax-2
p100	0.5	1.3	1.3	0.8	0.4	1.0	0.7
p52	0.1	1.2	1.1	0.7	0.4	0.4	0.2

Ratio (normalized to ACTB)	Jurkat	MT-2	Tesi	Mot	C3-44-Mo
p100	0.27	0.56	0.42	1.61	0.35
p52	0.02	0.53	0.63	1.36	0.67
Fascin	0.11	0.89	0.89	1.13	2.15

Figure 6 Heym et al.

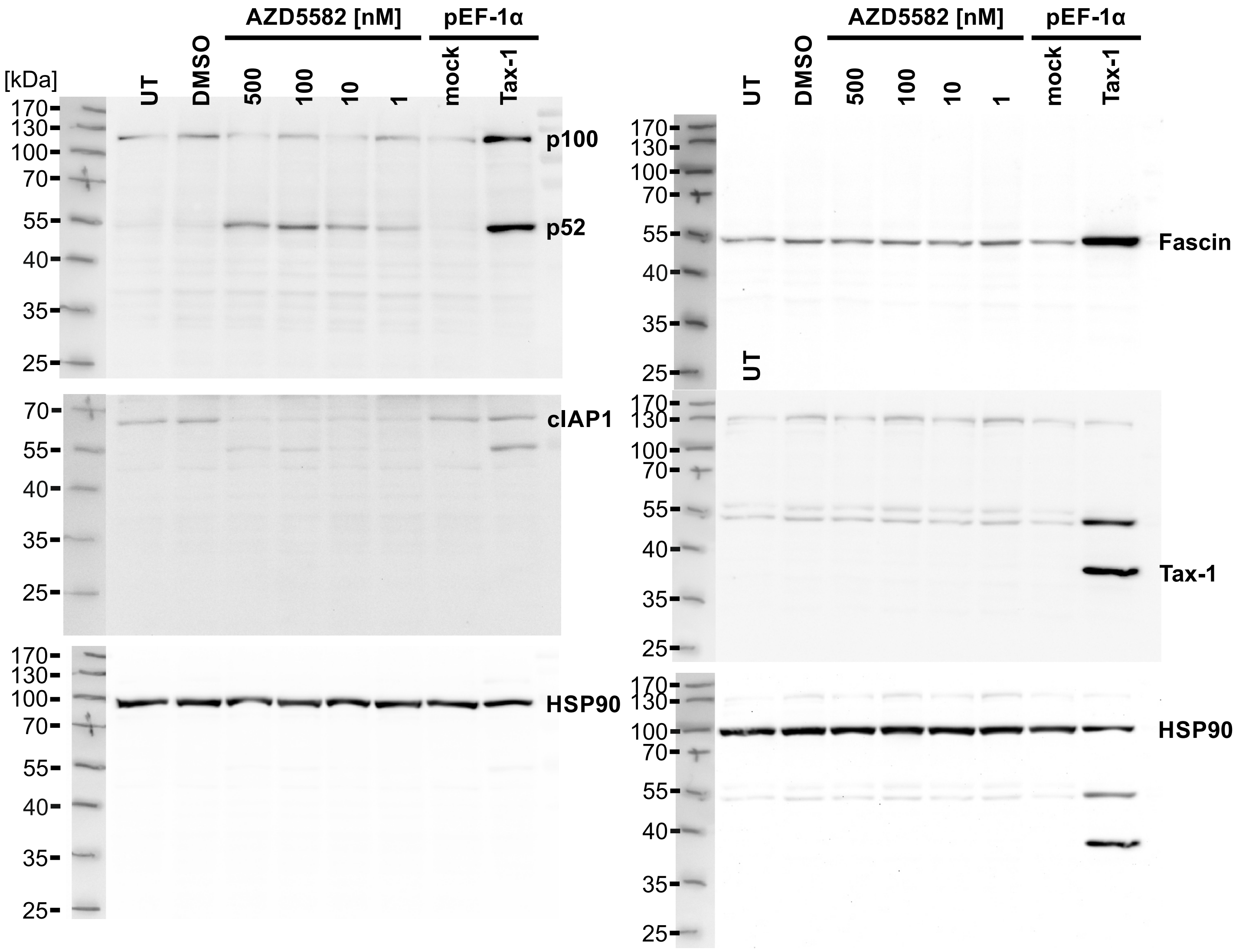

Ratio (normalized to ACTB)	UT	DMSO	AZD5582				mock	Tax-1
			500 nM	100 nM	10 nM	1 nM		
**p100**	0.22	0.35	0.16	0.26	0.13	0.26	0.16	0.97
**p52**	0.07	0.11	0.60	0.61	0.35	0.23	0.08	1.20
**cIAP1**	0.19	0.23	0.09	0.09	0.11	0.14	0.24	0.28
**Fascin**	0.35	0.42	0.43	0.38	0.37	0.48	0.40	1.99
**Tax-1**	0.00	0.00	0.00	0.00	0.00	0.00	0.00	1.69

Figure 7A Heym et al.

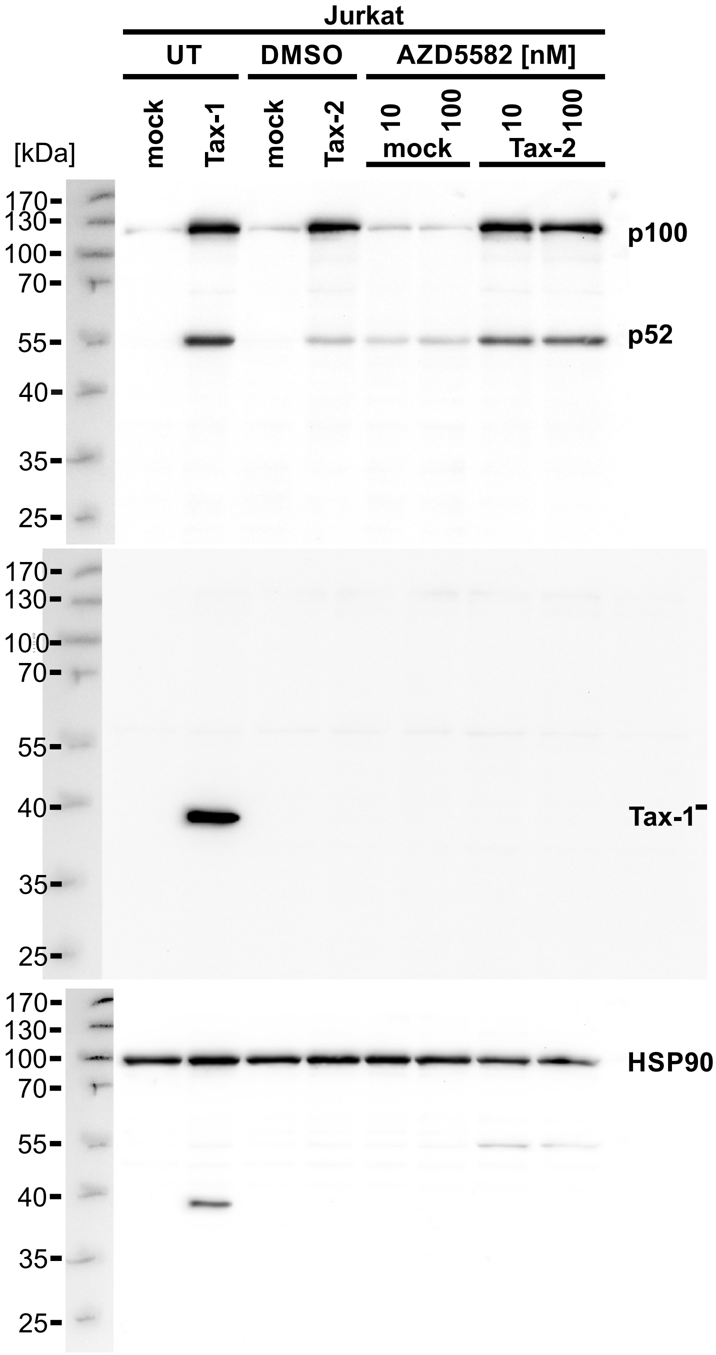

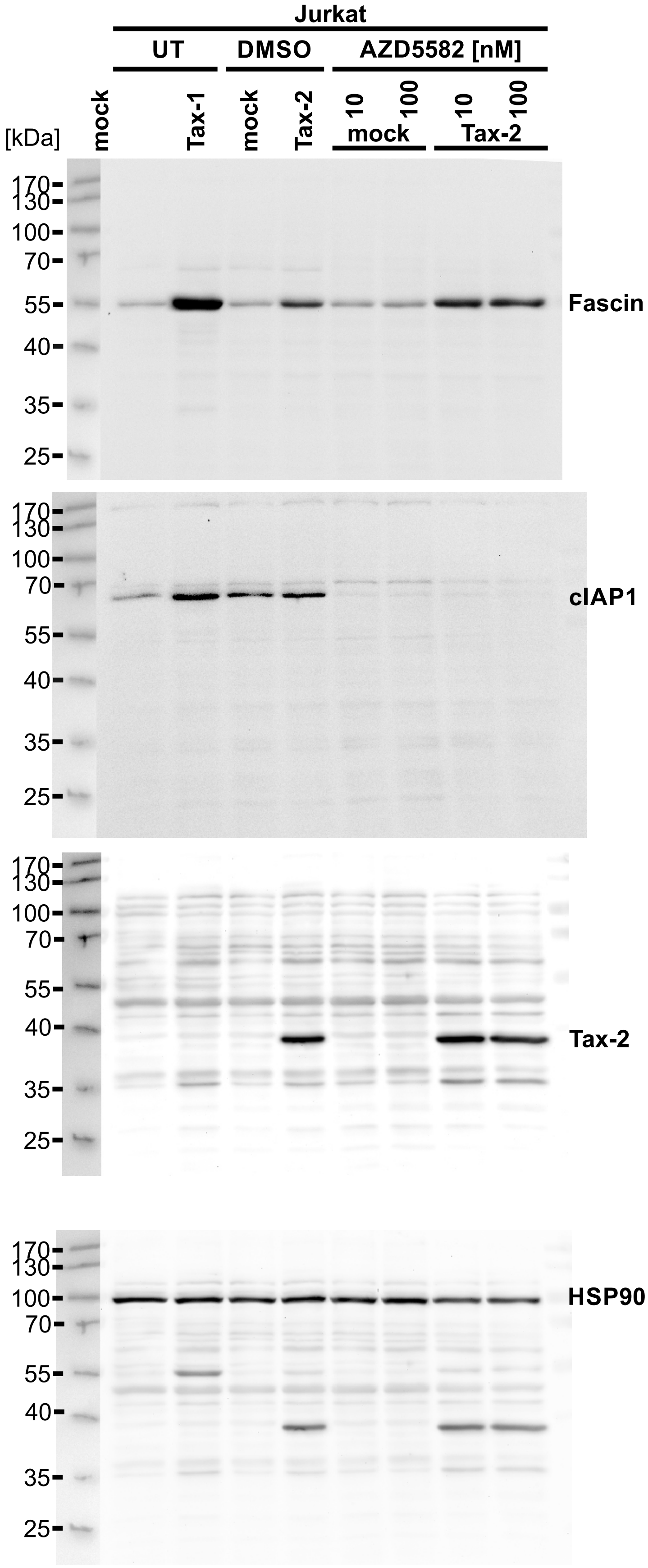

Ratio (normalized to ACTB)	Jurkat							
	UT		DMSO		AZD5582 [nM]			
					10	100	10	100
	mock	Tax-1	mock	Tax-2	mock	mock	Tax-2	Tax-2
**p100**	0.18	0.96	0.23	0.81	0.11	0.12	1.13	0.78
**p52**	0.09	1.45	0.14	0.35	0.41	0.56	1.31	1.19
**Tax-1**	0.00	2.03	0.01	0.00	0.00	0.00	0.00	0.00
**Fascin**	0.31	1.58	0.33	1.08	0.35	0.43	1.52	1.88
**cIAP1**	0.79	1.25	1.13	1.67	0.40	0.57	0.51	0.45
**Tax-2**	0.00	0.00	0.03	2.71	0.07	0.07	3.88	3.83

Figure 7D Heym et al.

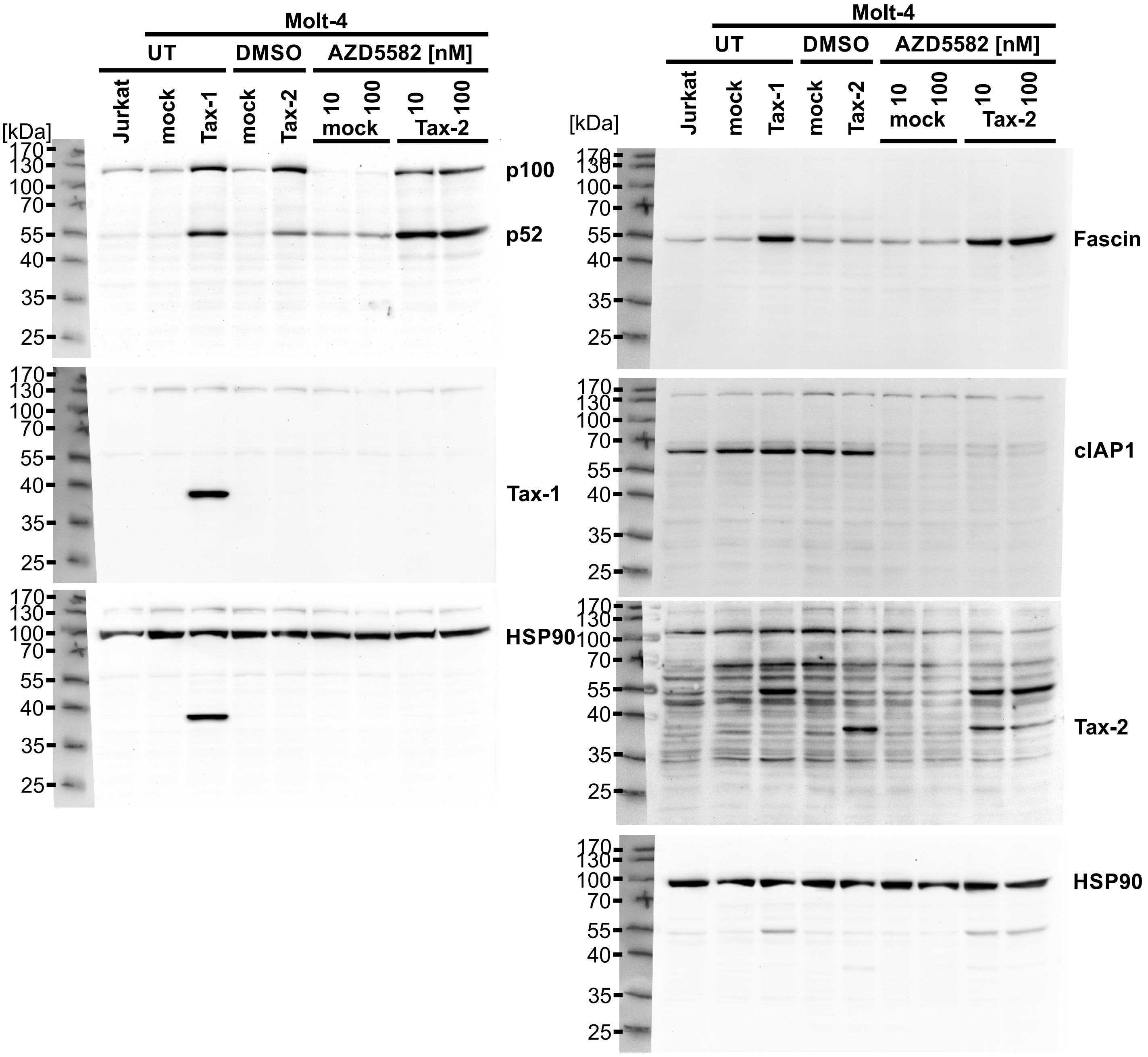

Ratio (normalized to ACTB)	Jurkat	Molt-4							
		UT		DMSO		AZD5582 [nM]			
						10	100	10	100
		mock	Tax-1	mock	Tax-2	mock	mock	Tax-2	Tax-2
**p100**	0.41	0.24	0.94	0.23	1.04	0.04	0.04	0.67	0.77
**p52**	0.09	0.07	0.92	0.12	0.51	0.27	0.32	1.20	1.13
**Tax-1**	2.70	0.03	0.00	0.04	0.02	0.00	0.00	0.00	0.00
**Fascin**	0.12	0.19	0.90	0.24	0.26	0.17	0.22	0.69	1.03
**cIAP1**	0.63	1.17	1.17	0.94	1.02	0.04	0.05	0.03	0.00
**Tax-2**	0.16	0.29	0.17	0.22	0.93	0.08	0.06	0.48	0.20

Apendix A2A Heym et al.

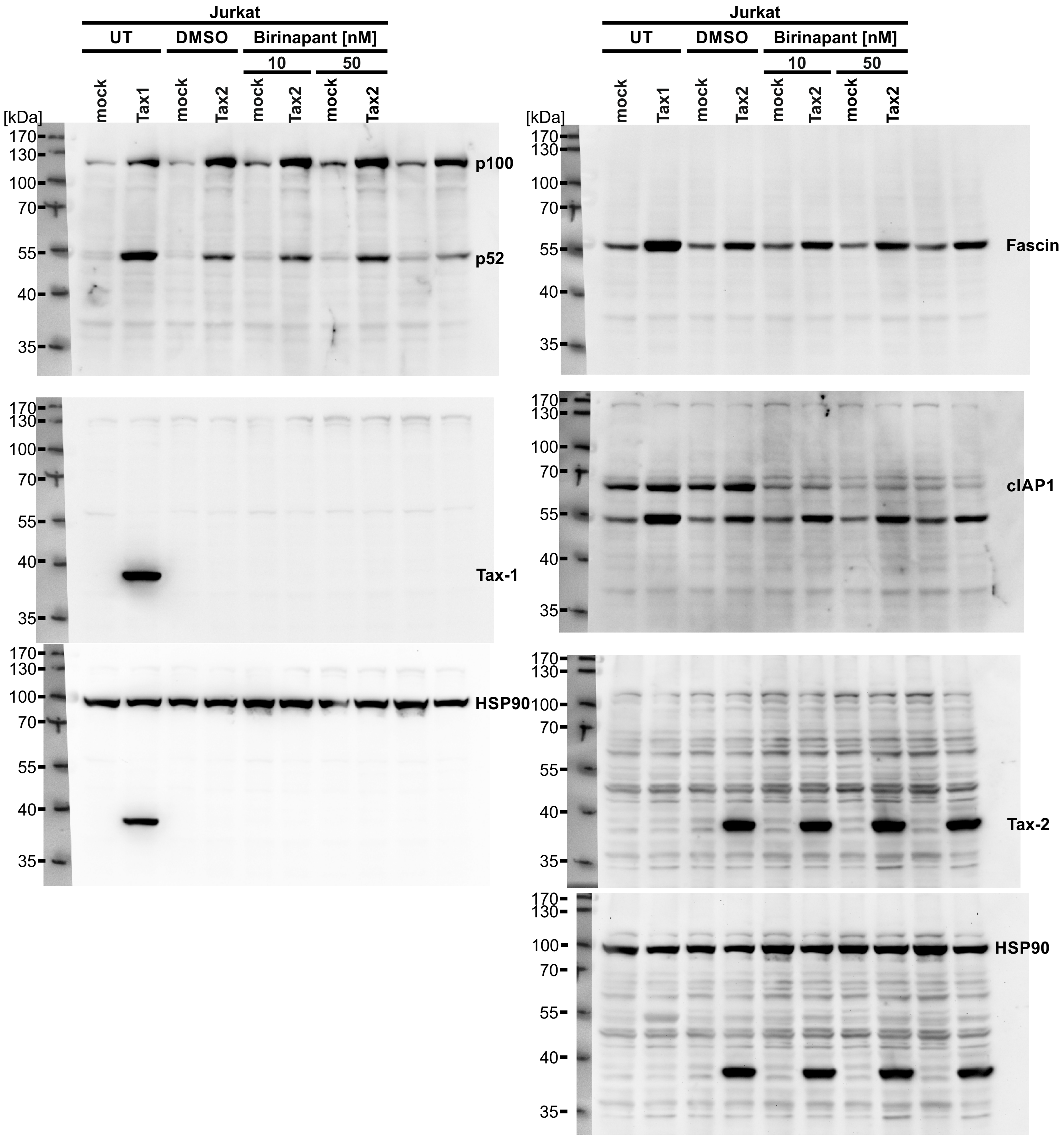

Ratio (normalized to ACTB)	Jurkat							
	UT		DMSO		Birinapant [nM]			
					10	10	100	100
	mock	Tax-1	mock	Tax-2	mock	Tax-2	mock	Tax-2
**p100**	0.20	0.77	0.33	1.16	0.43	1.25	0.45	1.23
**p52**	0.07	1.19	0.06	0.42	0.07	0.53	0.10	0.59
**Tax-1**	0.05	1.42	0.08	0.00	0.00	0.00	0.00	0.00
**Fascin**	0.13	1.08	0.31	0.71	0.36	0.77	0.47	0.78
**cIAP1**	0.67	0.96	0.65	1.00	0.40	0.45	0.20	0.44
**Tax-2**	0.03	0.07	0.22	1.35	0.28	1.49	0.20	1.54
